# Phylogenetically diverse *Bradyrhizobium* genospecies nodulate Bambara groundnut (*Vigna subterranea* L. Verdc) and soybean (*Glycine max* L. Merril) in the northern savanna zones of Ghana

**DOI:** 10.1093/femsec/fiac043

**Published:** 2022-04-11

**Authors:** Josephine A Adjei, Aregu A Aserse, Markku Yli-Halla, Benjamin D K Ahiabor, Robert C Abaidoo, Kristina Lindstrom

**Affiliations:** Department of Crop and Soil Sciences, Faculty of Agriculture, Kwame Nkrumah University of Science and Technology, PMB, Kumasi, Ghana; Faculty of Biological and Environmental Sciences, University of Helsinki, FIN-00014 Helsinki, Finland; Council for Scientific and Industrial Research, Savanna Agricultural Research Institute, PO Box 52, Tamale, Ghana; Faculty of Biological and Environmental Sciences, University of Helsinki, FIN-00014 Helsinki, Finland; Department of Agricultural Sciences, University of Helsinki, FIN-00014 Helsinki, Finland; Council for Scientific and Industrial Research, Savanna Agricultural Research Institute, PO Box 52, Tamale, Ghana; Department of Theoretical and Applied Biology, Kwame Nkrumah University of Science and Technology, PMB, Kumasi, Ghana; International Institute of Tropical Agriculture, PMB 5320, Ibadan, Nigeria; Faculty of Biological and Environmental Sciences, University of Helsinki, FIN-00014 Helsinki, Finland

**Keywords:** biological nitrogen fixation, *Bradyrhizobium*, multi-locus sequence analysis, housekeeping genes, Bambara groundnut, soybean

## Abstract

A total of 102 bacterial strains isolated from nodules of three Bambara groundnut and one soybean cultivars grown in nineteen soil samples collected from northern Ghana were characterized using multilocus gene sequence analysis. Based on a concatenated sequence analysis (*glnII-rpoB-recA-gyrB-atpD*-*dnaK*), 54 representative strains were distributed in 12 distinct lineages, many of which were placed mainly in the *Bradyrhizobium japonicum* and *Bradyrhizobium elkanii* supergroups. Twenty-four of the 54 representative strains belonged to seven putative novel species, while 30 were conspecific with four recognized *Bradyrhizobium* species. The *nodA* phylogeny placed all the representative strains in the cosmopolitan *nodA* clade III. The strains were further separated in seven *nodA* subclusters with reference strains mainly of African origin. The *nifH* phylogeny was somewhat congruent with the *nodA* phylogeny, but both symbiotic genes were mostly incongruent with the core housekeeping gene phylogeny indicating that the strains acquired their symbiotic genes horizontally from distantly related *Bradyrhizobium* species. Using redundancy analysis, the distribution of genospecies was found to be influenced by the edaphic factors of the respective sampling sites. In general, these results mainly underscore the high genetic diversity of Bambara groundnut-nodulating bradyrhizobia in Ghanaian soils and suggest a possible vast resource of adapted inoculant strains.

## Teaser

In the northern savanna zones of Ghana, Bambara groundnut-nodulating *Bradyrhizobium* species are taxonomically diverse.

## Introduction

Bambara groundnut (*Vigna subterranea L. Verdc*) is an indigenous grain legume that is cultivated across the entire continent of Africa and also in some parts of southeast Asia (Feldman *et al*. 2019). In Africa, it is, after cowpea, regarded as the second most important indigenous legume cultivated by farmers on smallholdings (Abu and Buah 2011, Jaiswal and Dakora 2019). Its annual world production is estimated at 330 000 tons, of which about half is produced in West Africa, mainly in Nigeria, Ghana, Togo and Benin (Directorate Plant Production 2011, Alhassan and Egbe 2013). In Ghana, Bambara is found growing in both the southern and northern parts of the country, but its production is more common in the northern savanna zones (Guinea and Sudan savanna agroecology) than in the southern regions (William *et al*. 2016). The crop does well in well-drained soils with a pH of 5.0–6.5 and thrives at an average temperature of 20–28°C and a mean annual rainfall of 500–600 mm (Directorate Plant Production 2011). Bambara groundnut is drought tolerant and can produce reasonable yields in marginal, low-input soils where other crops may fail (Feldman *et al*. 2019). Generally, Bambara groundnut is cultivated as landraces and it is featured either as a monocrop or an intercrop for cereals or root crops. The matured seeds provide good nutritional value that can help enhance the nutritional status of farm families (Feldman *et al*. 2019). Although largely underutilized, the crop holds great potential to address food insecurity especially in areas impacted or likely to be impacted by climate change.

Bambara groundnut, like other legumes, forms a symbiotic association with nitrogen-fixing soil bacteria commonly called rhizobia to provide fixed nitrogen (N) via biological nitrogen fixation (BNF) (Mohale *et al*. 2014). The BNF process is one of the essential ecosystem services that plays a vital role in agricultural systems by increasing grain legume productivity and helping to improve the carbon (C) and N content of soils (Jensen and Hauggaard-Nielsen 2003). Particularly in smallholder farming systems where access to synthetic N fertilizer is limited due to high cost and inadequate supply, BNF is essential (Houlton *et al*. 2019). Bambara groundnut can fix as much as 98% of its N nutrition by forming a symbiotic association with rhizobia (Sprent *et al*. 2010, Mohale *et al*. 2014). The main symbionts of this crop belong to the genus *Bradyrhizobium* (Grönemeyer and Reinhold-Hurek 2018). This genus is geographically widespread and its species have been isolated from different leguminous plant species in several countries and from different edaphoclimatic conditions (Helene *et al*. 2020). Validly published *Bradyrhizobium* species such as *B. pachyrhizi*,*B. vignae*,*B. yuanmingense*,*B. daqingense*,*B. huanghuaihaiense*,*B. iriomotense*,*B. jicamae*,*B. lablabi*,*B elkanii* and *B. subterraneum* are known symbionts of Bambara groundnut (Grönemeyer *et al*. 2014, 2015b, 2016, Puozaa *et al*. 2017, Ibny *et al*. 2019). Although vital for increased food production in Africa, the diversity and distribution of these Bambara groundnut-associated microsymbionts have not been well studied. Thus, more studies are needed to uncover their diversity in African soils and their potential for an adapted inoculant production. In addition, strain identification and characterization would improve knowledge of their contribution to ecosystem functioning and thus provide information on factors that shape their diversity and distribution (Jaiswal and Dakora 2019). This information would contribute toward the understanding of adaptational properties important to the suitability of a strain for use in inoculants at a particular geographical location (Grönemeyer and Reinhold-Hurek 2018).

Soybean plays an important role in the diets of many households due to its high nutritional value. The crop is cultivated under diverse climatic conditions worldwide and accounts for 80% of the land area used for legume production in the world (Herridge *et al*. 2008). Although native to northeast Asia, the crop is increasingly becoming an important cash crop in Africa with countries like South Africa, Zambia, Nigeria and Uganda being lead producers (Khojely *et al*. 2018). In Ghana, ∼80% of the crops’ national production, estimated at 182 000 tons, takes place in the northern savanna zones (Aidoo *et al*. 2013). The crop is mainly cultivated for food and income and the haulms are used as feed for livestock. Soybean is capable of hosting diverse groups of rhizobia in root nodules to meet its large N requirement for optimum growth. Rhizobial species belonging to the genera *Bradyrhizobium*,*Rhizobium*,*Mesorhizobium* and *Ensifer*(*Sinorhizobium*) are capable of forming effective N_2_-fixing symbioses with soybean (Chen *et al*. 1995, Saldaña *et al*. 2003). Diverse *Bradyrhizobium* species, including *B. diazoefficiens* (Delamuta *et al*. 2013),*B. daqingense* (Wang *et al*. 2013a),*B. elkanii* (Kuykendall *et al*. 1992),*B. huanghuaihaiense* (Zhang *et al*. 2012),*B. japonicum* (Jordan 1982),*B. liaoningense* (Xu *et al*. 1995) and *B. ottawaense* (Yu *et al*. 2014), have been isolated from soybean nodules. Previously, soybean was regarded as fairly specific with regard to its requirement for particular rhizobia, but breeding programs have tackled this problem with the release of improved varieties that are promiscuous and can nodulate with indigenous rhizobial population (Abaidoo *et al*. 2000, Mpepereki *et al*. 2000). Despite the increasing cultivation of soybean in Ghana, very little information is available on the diversity of indigenous rhizobia that nodulate this legume in Ghanaian soils. Thus, to guide the exploration of the indigenous rhizobial population for strains suitable to soybean inoculant production, studies on the diversity of soybean-nodulating species in Ghanaian soils are needed.

Molecular and genetic methods have been used to effectively study the taxonomy and diversity of rhizobia (Jaiswal *et al*. 2016). For identification of rhizobial genotypes, protein-coding housekeeping genes, such as *recA*, have been demonstrated by several authors as a good phylogenetic marker (Aserse *et al*. 2012a, Jiao *et al*. 2015, Mousavi *et al*. 2015, Tena *et al*. 2017, Asfaw *et al*. 2020). However, individual housekeeping genes may have different evolutionary rates or histories among bacterial taxa and hence the phylogeny of a single gene may not provide a perfect snapshot of the evolution of a species (Peter *et al*. 1996; Tian *et al*. 2012). Thus, a phylogeny based on multilocus sequence analysis (MLSA) involving several housekeeping genes can provide a more accurate reflection of diversity and taxonomy of rhizobial species (Glaeser and Kämpfer 2015).

We aimed to obtain a general understanding of the presence, taxonomy, ecological distribution and diversity of Bambara groundnut- and soybean-nodulating rhizobia in the soils of the northern savanna zones of Ghana. To achieve this, we isolated rhizobia from nodules of Bambara groundnut and soybean sown in potted soils sampled from the Guinea and Sudan savanna agroecological zones of Ghana. A total of 102 rhizobial strains were recovered from the trapping experiment and these were screened based on phylogenetic analysis of the *recA* gene. Primarily, considering isolation sites and phylogenetic groups, 54 strains representing diverse clusters were then used in MLSA of six protein-coding housekeeping genes, including genes coding for recombination protein (*recA*), glutamine synthetase II (*glnII*), RNA polymerase beta subunit (*rpoB*), DNA gyrase subunit B (*gyrB*), ATP synthase subunit beta (*atpD*) and the 70kDa chaperone (*dnaK*). To further refine adaptational properties, the phylogeny of two accessory genes, *nodA* encoding *N*-acyltransferase required for nodulation and *nifH* that encodes dinitrogenase reductase required for N_2_, was determined. The nodulation capacity of the representative strains was studied in a growth chamber using Bambara groundnut as trap crop. The physicochemical properties of the sampling sites were also examined to assess their influence on the distribution and composition of the genospecies.

## Materials and methods

### Soil sampling and characterization

The isolates used in the study were trapped from soils sampled from nineteen farmers’ fields located in the Guinea and Sudan savanna agroecological zones of Ghana. These agroecological zones are warm, semi-arid environments with a unimodal rainfall pattern that occurs between May and October. The vegetation consist of short deciduous fire-resistant trees that include anthropic species with open canopies, such as neem (*Azadirachta indica*), shea (Vitellaria paradoxa), mango (*Mangifera indica*), baobab (Adansonia digitata), silk-cotton (Ceiba* petandra*), African mahogany (Khaya senegalensis) and locust bean (Parkia biglobosa) trees. The ground flora comprises various grass species of varying height. The fields had a cropping history of maize cultivation and the soils according to the World Reference Base classification system (IUSS Working Group WRB 2015) represent seven soil types (Table S1, Supporting Information). The soil samples were analyzed for physicochemical characteristics mainly according to Van Reeuwijk (2002). Soil pH was measured in 1:2.5 soil/water (w/v) suspension. Organic carbon (C) was determined by the Walkley and Black wet combustion method and N by the Kjeldahl method. Plant-available P was extracted by the Bray 1 method (0.03 M NH_4_F–0.025 M HCl) and measured with a spectrophotometer using the molybdate blue method. Exchangeable Ca, Mg, K, Na, Mn and Zn were extracted with 1.0 M ammonium acetate buffered at pH 7 and exchangeable acidity was determined by saturating samples with unbuffered 1.0 M KCl solution titrated with 0.02 M NaOH. Results were expressed as cmol_(+)_ kg^–1^. Mn, Zn, Ca and Mg were measured with an atomic absorption spectrophotometer and K and Na, with a flame photometer. Because the soils were nonsaline, the effective cation exchange capacity (CEC_ef_) was calculated as the sum of exchangeable Ca, Mg, K and Na, and the base saturation (BS) was calculated as BS = 100 × (Ca+Mg+K+Na)/CEC_ef_, respectively. Particle size distribution was determined with the hydrometer method.

### Trapping, isolation and authentication of rhizobia nodulation ability

For the trapping experiment, seeds of three Bambara groundnut landraces with different seed coat colors (wine, cream and black) and one improved variety of soybean (Favor) were obtained, respectively, from markets in northern Ghana and the Council for Scientific and Industrial Research–Savanna Agricultural Research Institute (CSIR–SARI), Tamale, Ghana. Four-liter capacity pots were filled with 3 kg of soil and arranged in a screen house at CSIR–SARI. The seeds of each genotype were separately surface sterilized with 70% ethanol for 1 min, followed by a treatment with 3% sodium hypochlorite for 4 min and then rinsed thoroughly in several changes of sterile distilled water (Somasegaran and Hoben 2012) and planted singly in each soil. The seedlings were supplied with water every other day or when required. Nodules were sampled after 45 days of plant growth; at ∼50% flowering stage, washed and stored temporally on silica gel. The desiccated nodules were rehydrated in sterile distilled water for about an hour after which they were surface sterilized first using 70% ethanol for 1 min, then 3% sodium hypochlorite for ∼3 min where after the nodules were rinsed in several changes of sterile distilled water. Each surface sterilized nodule was crushed in 100 µl sterile distilled water after which 50 µl was spread on yeast mannitol agar (YMA) plates containing Congo red (Somasegaran and Hoben 2012). The plates were incubated at 28–30°C for up to 10 days depending on the growth habit of each isolate. Colonies typical of rhizobia were repeatedly streaked (Somasegaran and Hoben 2012) until pure cultures were obtained. The pure cultures were then stored in yeast extract mannitol (YEM) broth containing 25% (v/v) glycerol at −20°C.

The nodulation ability of 54 representative bacterial isolates (52 isolates from Bambara groundnut and two isolates from soybean) was tested using the Bambara groundnut landraces used in the trapping experiment under controlled conditions in a growth chamber at the University of Helsinki. The Bambara groundnut was selected for this screening because of its promiscuous nodulation pattern. The seeds were surface sterilized as previously described (Somasegaran and Hoben 2012) and germinated on 0.75% (w/v) water agar in Petri dishes. The plates were incubated at 28°C until radicles were ∼2 cm long and then the seedlings were transferred aseptically into plastic growth pouches measuring 16.5 cm by 35 cm (CYG seed germination pouch; Mega International), containing sterile N-free nutrient solution (Jensen 1942) and arranged on holders. Bacterial cultures grown to log phase (1 ml) were inoculated on the base of the seedlings after 3 days and then grown in a growth chamber with a 14 h light period (22°C for 1 h, 25°C for 12 h and 2°C for 1 h) and a 10 h dark period at 18°C. The plants were watered with quarter-strength N-free nutrient solution once a week or when necessary, with sterile Milli-Q (Helsinki, Finland) water.. The plants were harvested after 35 days for root nodule observation. The nodulation was recorded by the presence of nodules and their N_2_-fixation efficiency was estimated by a pink or red coloration inside the nodules.

### DNA extraction, PCR amplification and sequencing

Extraction of genomic DNA from bacterial cultures grown in YEM until late log phase was carried out using NucleoSpin Genomic DNA extraction kit following the manufacturer's instruction (MACHEREY-NAGEL, Inc.). The integrity and purity of the DNA were checked using 1.5% (w/v) agarose gel electrophoresis. The genomic DNA of the isolates was stored at −20°C. The gene encoding recombinase A (*recA*) was amplified using the primers TSrecAF (CAACTGCMYTGCGTATCGTCGAAGG) and TSrecAR (CGGATCTGGTTGATGAAGATCACCATG) (Stępkowski *et al*. 2005). The polymerase chain reaction (PCR) reaction mixture was formulated according to the instruction of the manufacturer (Finnzymes): a 50 µl reaction mixture containing 1 µl DNA template, 5× Phusion HF buffer (1×), dNTPs (200 µM), primers (0.5 µM each) and Phusion DNA polymerase (0.01 U/µl). The PCR reaction was done at an initial denaturation of 98°C for 3 min followed by 35 cycles of denaturation at 98°C for 15 s, annealing at 68°C for 30 s and elongation at 72°C for 30 s. A final elongation was then done at 72°C for 10 min and the samples were held temporarily at 4°C. The quality of the PCR products was checked using 1.5% (w/v) agarose gel electrophoresis after which they were sequenced at the Institute of Biotechnology, University of Helsinki by Sanger technology using same primers used for the PCR amplification.

### Sequence data analyses

The quality of the *recA* sequences was checked using the Gap4 program as implemented in the Staden package 2.0.0 (Staden *et al*. 2000). The sequences of the representative isolates were compared with sequences of reference strains in the National Center for Biotechnology Information (NCBI) GenBank database using the standard nucleotide Basic Local Alignment Search Tool (BLASTn) program (https://blast.ncbi.nlm.nih.gov/Blast.cgi; Altschul *et al*. 1990). Sequences of closely related reference strains were aligned with the sequences of the 102 rhizobial isolates using CLUSTAL W in MEGAX (Larkin *et al*. 2007). ModelFinder (Kalyaanamoorthy *et al*. 2017) embedded in IQ-TREE was used to determine a best-fit model according to the Bayesian Information Criterion (BIC) and the *recA* phylogeny was inferred using the maximum likelihood (ML) method (Nguyen *et al*. 2015). Branch support was assessed with the SH-like approximate likelihood ratio test (SH-aLRT) (Guindon *et al*. 2010) and ultrafast (UF) bootstrap integrated in IQ-TREE using 1000 replicates (Hoang *et al*. 2018). A cluster/lineage was considered probable when both SH-aLRT and UF bootstrap values are ≥80% and ≥95%, respectively. The pairwise sequence similarity between the references and representative strains was determined using the BLASTn program (align two or more sequences) (https://blast.ncbi.nlm.nih.gov/Blast.cgi?PROGRAM=blastn&BLAST_SPEC=GeoBlast&PAGE_TYPE=BlastSearch). For this sequence similarity analyses, we used the sequence used for making the alignments and for phylogenetic trees.

Based on the obtained *recA* phylogenetic tree, 54 representative strains were selected from the different clusters mainly considering isolation sites and phylogenetic group. The selected strains were then paired-end sequenced (2 × 150 bp) using Illumina NextSeq500 platform at the Institute of Biotechnology, University of Helsinki. For the purpose of this study, the reads were aligned with reference sequences in the GenBank and then additional core (*glnII*, *gyrB*, *rpoB*, *atpD* and *dnaK*) and symbiotic (*nodA* and *nifH*) genes were retrieved for further phylogenetic analyses. The individual gene sequences of the representative strains together with all available relevant reference strains retrieved from the NCBI GenBank database were aligned using CLUSTAL W. The six housekeeping genes were concatenated using Sequence Matrix (Vaidya *et al*. 2011). A best-fit model according to BIC was determined by the ModelFinder for all single gene alignments and the edge-proportional partition model (Chernomor *et al*. 2016) was used for the concatenated gene alignment. Phylogenetic trees were constructed from the single gene alignments and the six concatenated housekeeping gene sequences (*recA-glnII-gyrB-atpD-rpoB-dnaK*) following a similar procedure as described above. The *recA*, *glnII*, *rpoB*, *gyrB*, *atpD*, *dnaK*, *nodA* and *nifH* gene sequences were subsequently submitted to the NCBI GenBank database where they received the accession numbers MW798381–MW798434, MW798435–MW798487, MW798488–MW798541, MW798542–MW798595, MW798596–MW798649, MW798650–MW798703, MW853620–MW853661 and MW867251–MW867298, respectively. The accession numbers of the reference sequences are provided in Table S4 (Supporting Information).

### Statistical analysis

Redundancy analysis (RDA) was used to examine the relationship between soil factors and the distribution of genospecies and *recA* clusters (Table S3, Supporting Information) identified in the study using the R program. The soil variables were first transformed (log X + 1) and normalized to overcome the different measurement units (Clarke and Gorley 2001). Multivariate analyses of variance (MANOVA) was performed to determine the presence of significant differences between soil properties of the sampling sites (Bates 2005). The soil variables that explained variations significantly between genospecies and *recA* clusters were determined using the ‘ordistep’ (forward selection) method embedded in the ‘vegan’ package of the R software. The significance level for assessing differences was set at 5% and a permutation test (permutation = 999) was done. To avoid multicollinearity effects, all highly correlated or repetitive variables were removed but one and the fitted model was selected accordingly to visualize the resulting RDA plot (Bates 2005, Borcard *et al*. 2011).

## Results

### Soil properties of the sampling sites

The physicochemical properties of the soils are presented in Table 1. Sand was the dominant fraction by 50–69% in all study soils, and the textural classes comprised sandy loam (11 soils, moderately coarse-textured), sandy clay loam (six soils, moderately fine-textured) and loamy sand (two soils, coarse-textured). The soils were acidic, the pH (H_2_O) covering a range from 4.5 to 6.2, where four soils had a pH below 5.0 and only one had a pH above 6. The low pH indicates that the soils were noncalcareous. Organic carbon content was rather low, ranging from 0.4% to 1.8%, mean 0.8%. The CEC_ef_ varied from 3.8 to 18.2 cmol_(+)_ kg^–1^ with 16 soils having a CEC_ef_ <10 cmol_(+)_ kg^–1^. The CEC_ef_ correlated closely with C content (*r* = 0.87, *P*< 0.001), suggesting that organic matter probably provided much of the CEC sites, while the correlation between CEC_ef_ and clay content was lower (*r* = 0.57, *P* = 0.0135). When calculated on the basis of CEC_ef_, and using the assumption that organic matter ( = 1.724 x organic C) has a CEC of 200 cmol kg^–1^, clay had an average CEC of 26 cmol kg^–1^ (range 9–42 cmol kg^–1^). Even though a few soils may also have contained some high-activity clays, the soils were mostly dominated by low-activity clay minerals, likely kaolinite. This conclusion is also supported by the soil classification. Electrical conductivity was not determined but the very low concentration of Na in most soils suggests that the soils were nonsaline and non-sodic. As usual, exchangeable Al correlated closely (*r* = −0.91, *P*< 0.0001) with soil pH. The most acidic soils (Manga, Upando, Golinga and Kunfabiala) had a base saturation between 64% and 78%, calculated from the CEC_ef_, suggesting a chance of a moderate Al toxicity. In the other soils, the base saturation was >83% with a low probability of Al toxicity. The variation of plant-available P covered a range from very low (0–3 mg kg^–1^) to high (>20 mg kg^–1^) concentrations, most soils falling into the low (3–11 mg kg^–1^) category, and only one soil (Golinga) was in a very low range (<3 mg kg^–1^). On the other hand, all the three soils, Bamahu, Tanina and Gunayili, fell in the high category with P concentrations <30 mg kg^–1^, thus being not excessively rich in plant-available P (Table 1).

**Table 1.** Physicochemical parameters of soil sampling sites.

		(%)				(%)			cmol_(+)_ kg^–1^						mg kg^–1^			
Location	pH (H_2_O)	Clay	Silt	Sand	Texture	OC	TN	BS	Ca	Mg	K	Na	CEC	Ex. Al+H	P Bray 1	Fe	Mn	Zn
Tansia	5.2	14	4	82	SL	0.76	0.06	85	3.31	1.92	0.33	0.03	6.54	0.95	6.90	1.160	7.26	0.42
Kasiesa	5.5	16	10	74	SL	0.72	0.06	89	5.02	2.78	0.16	0.09	9.00	0.95	3.70	0.002	5.15	2.52
Bogrigo	5.5	26	16	58	SCL	0.96	0.09	95	6.41	4.27	1.03	0.05	12.37	0.62	14.90	5.135	34.4	0.66
Belempiisi	5.1	12	6	82	LS	0.56	0.04	77	2.14	1.60	0.05	0.01	4.95	1.15	4.30	0.002	3.43	0.26
Manga	4.5	28	22	50	SCL	0.40	0.03	64	1.49	0.85	0.09	0.01	3.79	1.35	12.2	0.002	0.69	0.28
Golinga	4.9	10	23	67	SL	0.56	0.04	78	2.56	1.49	0.10	0.08	5.44	1.2	2.00	2.445	0.49	0.28
Upando	4.7	22	16	62	SCL	0.56	0.05	77	2.46	1.92	0.04	0.02	5.74	1.3	3.3	0.002	45.64	0.26
Masaka	5.0	26	20	54	SCL	0.92	0.06	86	4.16	2.99	0.22	0.01	8.60	1.22	7.3	3.650	49.86	0.88
Changnaayili	5.2	18	10	72	SL	1.00	0.08	88	4.38	3.20	0.12	0.09	8.89	1.1	4.6	0.180	27.92	0.56
Mepasem	5.9	18	6	76	SL	0.76	0.08	90	3.74	2.56	0.72	0.02	7.79	0.75	4.2	1.245	60.04	0.99
Gunayili	6.2	30	20	50	SCL	1.80	0.11	99	9.61	8.11	0.20	0.03	18.21	0.25	26	4.430	65.64	0.25
Achubonyor	5.9	16	8	76	SL	0.68	0.04	88	3.10	2.14	0.14	0.00	6.13	0.76	6.1	2.215	26.27	1.48
Nabori	5.4	20	14	66	SL	0.84	0.06	89	3.63	2.56	0.18	0.01	7.13	0.75	10.3	0.002	59.16	0.27
Loho	5.9	14	8	78	SL	0.52	0.09	83	2.46	1.49	0.07	0.01	4.82	0.8	3.3	0.015	4.19	0.88
Tanina	5.1	10	4	86	LS	0.60	0.03	78	2.24	1.60	0.10	0.07	5.16	1.15	24.8	0.002	6.71	0.74
Bamahu	5.9	20	12	68	SL	1.28	0.09	92	4.80	4.16	0.34	0.06	10.14	0.77	29.4	5.100	16.97	1.24
Gbanko	5.1	22	14	64	SCL	1.40	0.09	86	3.74	2.78	0.00	0.35	8.40	1.18	5.4	9.270	33.88	0.25
Sanwana	5.8	18	6	76	SL	0.92	0.06	91	3.84	2.67	0.20	0.01	7.38	0.65	6.3	5.285	12.94	0.95
Kunfablala	4.7	18	8	74	SL	0.60	0.06	75	2.14	1.28	0.23	0.01	4.91	1.25	5.7	5.250	15.86	6.61

CEC, cation exchange capacity; Ex. Al+H, exchangeable acidity; BS, base saturation; OC, organic carbon; SL, sandy loam; SCL, sandy clay loam; LS, loamy sand.

### Bacterial isolates

A total of 102 bacterial isolates sampled from 19 locations in the Guinea and Sudan savanna agroecological zones of Ghana were recovered from root nodules of three Bambara groundnut landraces: 35 isolates from the wine seed-coated race, 28 isolates from the cream seed-coated race and 32 isolates from the black seed-coated race, while the remaining seven isolates associated with the soybean variety, Favor. All the isolates obtained showed growth characteristic of *Bradyrhizobium*; they were slow growing and showed an alkaline reaction on YMA plates containing bromothymol blue. Of the 54 representative isolates selected for authentication, 44 were able to effectively nodulate Bambara groundnut. The distribution of the isolates with respect to the sampling sites and information on nodulation are shown in Table S1 (Supporting Information) and Table 2, respectively.

**Table 2.** Taxonomic affiliation, genospecies groups and nodulation status of bradyrhizobial strains identified in this study.

Strain	Closest type species	Nucleotide identity (%)	Genospecies	*recA*	*rpoB*	*glnII*	*gyrB*	*atpD*	*dnaK*	*nodA*	*nifH*	nod	fix
BGE11C2	*B. yuanmingense*	98.9	I	I	I	I	I	I	I	III.3d	1	+	+
BGE13P3	*B. yuanmingense*	96.9	I	III	I	VIII	I	XI	I	III.3d	1	+	+
BGE21P8	*B. yuanmingense*	96.2	I	IV	I	VII	I	IV	I	*	*	+	+
BGN18P1	*B. yuanmingense*	96.6	I	IV	I	II	I	II	I	III.3a	4	+	+
BGN2B7	*B. yuanmingense*	96.5	I	IV	I	II	I	II	I	III.3a	4	+	+
BGN2C1	*B. yuanmingense*	96.7	I	IV	I	II	I	II	I	III.3a	4	+	+
BGS3B1	*B. yuanmingense*	96.6	I	IV	I	II	I	II	I	III.3a	4	+	+
BGW10P5	*B. yuanmingense*	96.8	I	IV	I	VII	I	XI	I	III.3a	*	−	−
BGW10P6	*B. yuanmingense*	96.5	I	IV	I	II	I	II	I	III.3a	4	+	+
BGW8B5	*B. yuanmingense*	96.6	I	IV	I	II	I	II	I	III.3a	4	+	+
BGW8P8	*B. yuanmingense*	96.6	I	IV	I	II	I	II	I	III.3a	4	+	+
SNFM2	*B. yuanmingense*	96.4	I	IV	I	VII	I	IV	I	III.3e	2	+	+
BGS17B2	*B. yuanmingense*	96.2	XII	II	II	I	VII	III	IV	III.3e	2	+	+
BGW19B2	*B. yuanmingense*	96.3	XII	II	II	I	VII	III	IV	III.3e	2	+	+
BGS17C2	*B. yuanmingense*	96.5	XII	IV	II	I	IX	XI	IV	III.3e	2	+	+
BGS17P1	*B. yuanmingense*	96.1	XII	IV	II	I	VII	XI	IV	III.3e	2	+	+
BGE11C3	*B. huanghuaihaiense*	94.8	II	VIII	III	V	II	I	II	III.3a	3	+	+
BGE13P2	*B. huanghuaihaiense*	94.8	II	VIII	III	V	II	I	II	III.3b	3	+	+
BGN18P3	*B. huanghuaihaiense*	94.8	II	VIII	III	V	II	I	II	III.3b	3	+	+
BGS3P2	*B. huanghuaihaiense*	94.8	II	VIII	III	V	II	I	II	III.3b	3	+	+
BGW10P7	*B. huanghuaihaiense*	95.3	II	VIII	III	V	II	VII	III	III.3b	3	+	+
SWFL2	*B. huanghuaihaiense*	94.7	II	VIII	III	V	II	I	II	III.3c	3	+	+
BGW7B4	*B. huanghuaihaiense*	94.7	II	VIII	III	V	II	I	II	III.3b	3	+	+
BGS3B6	*B. huanghuaihaiense*	95.0	III	V	IV	VI	IV	V	I	*	*	−	−
BGN1C6	*B. guangxiense*	97.0	IV	XI	V	IX	VI	VI	I	*	6	−	−
BGW8P7	*B. guangxiense*	97.0	IV	XI	V	IX	VI	VI	I	*	6	−	−
BGW20C3	*B. arachidis*	96.1	V	VII	III	IV	VIII	VII	III	*	*	−	−
BGE14P2	*B. vignae*	99.8	VII	VI	VII	VIII	III	XII	VII	III.3a	4	+	+
BGE14P7	*B. vignae*	99.7	VII	VI	VII	VIII	III	XII	VII	*	*	+	+
BGN1C1	*B. vignae*	99.6	VII	VI	VII	VIII	III	XII	VII	*	3	−	−
BGN1P6	*B. vignae*	99.4	VII	VI	VII	VIII	III	XII	VII	III.3a	4	+	+
BGN2P1	*B. vignae*	99.3	VII	VI	VII	VIII	III	XII	VII	III.3a	4	+	+
BGS16B1	*B. vignae*	99.2	VII	VI	VII	VIII	III	XII	VII	III.3a	4	+	+
BGS16C5	*B. vignae*	99.4	VII	VI	VII	VIII	III	XII	VII	III.3a	4	+	+
BGS4B1	*B. vignae*	99.8	VII	VI	VII	VIII	III	XII	VII	III.3a	4	+	+
BGW19C5	*B. vignae*	99.3	VII	VI	VII	VIII	III	XII	VII	*	4	−	−
BGW20C1	*B. vignae*	99.3	VII	VI	VII	VIII	III	XII	VII	III.3a	4	+	+
BGW9C3	*B. vignae*	99.3	VII	VI	VII	VIII	III	XII	VII	III.3a	4	+	+
BGE21P1	*B. embrapense*	95.0	VIII	XII	VIII	X	XI	XIII	VIII	III.3g	5	+	+
BGE22B2	*B. embrapense*	95.1	VIII	XII	VIII	X	XI	XIII	VIII	III.3g	5	+	+
BGE22C2	*B. embrapense*	95.2	VIII	XII	VIII	X	XI	XIII	VIII	III.3g	5	+	+
BGN18B1	*B. embrapense*	95.2	VIII	XII	VIII	X	XI	XIII	VIII	III.3g	5	+	+
BGN18C1	*B. embrapense*	95.2	VIII	XII	VIII	X	XI	XIII	VIII	III.3g	5	+	+
BGN2B2	*B. embrapense*	95.2	VIII	XII	VIII	X	XI	XIII	VIII	III.3g	5	+	+
BGN2C9	*B. embrapense*	95.1	VIII	XII	VIII	X	XI	XIII	VIII	III.3g	5	+	+
BGN6P7	*B. embrapense*	95.2	VIII	XII	VIII	X	XI	XIII	VIII	III.3g	5	+	+
BGW7P5	*B. embrapense*	95.3	VIII	XII	VIII	X	XI	XIII	VIII	III.3g	5	+	+
BGE22B1	*B. mercantei*	95.9	IX	XIII	IX	XI	XII	XIV	IX	III.3f	5	+	+
BGN6P8	*B. mercantei*	95.9	IX	XIII	IX	XI	XII	XIV	IX	III.3f	5	+	+
BGS3C3	*B. mercantei*	95.7	IX	XIII	IX	XI	XII	XIV	IX	III.3f	5	+	+
BGW7B2	*B. mercantei*	95.4	IX	XIII	IX	XI	I	XIV	IX	III.3a	5	+	+
BGN2P8	*B. denitrificans*	91.2	X	IX	X	XII	XIV	XI	V	*	7	−	−
BGW20C2	*B. denitrificans*	91.9	XI	X	XI	XIII	XIII	X	VI	*	8	−	−

The *recA* clusters are based on the 54 representative strains in Fig. S6 (Supporting Information).

Nod, nodulation; fix, nitrogen fixation; *, genes absent; −, no nodulation and/or nitrogen fixation.

### Identification and single gene phylogenies

**Figure 1.**
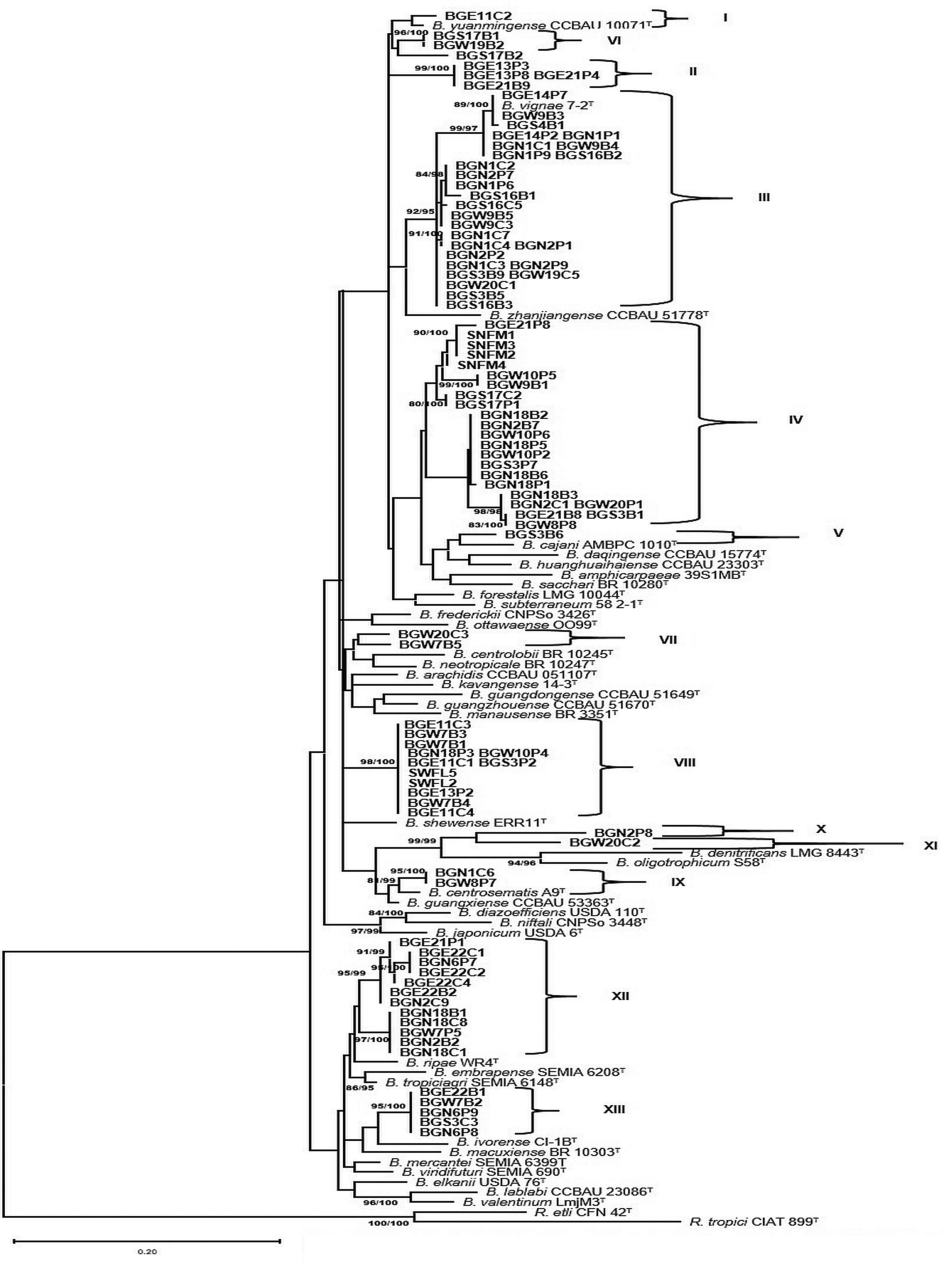
Rooted maximum likelihood phylogenetic tree based on *recA* gene showing relationship among the new isolates and reference strains in the genus *Bradyrhizobium*. SH-aLRT and ultrafast bootstrap values were inferred from 1000 replicates and are indicated at the nodes when SH-aLRT value is ≥80% and ultrafast value is ≥95%. The scale bar indicates the estimated nucleotide substitution rate. *R. tropici* CIAT 899^T^ and *R. etli* CFN 42^T^ were used as outgroup.

Based on the *recA* gene sequence (375 bp) comparisons, all 102 isolates were identified as *Bradyrhizobium* spp. These strains were separated into thirteen phylogenetic clusters designated as clusters I–XIII (Fig. 1) and shared a sequence similarity of ≥96% within each group (data not shown). The clusters II, III, IV, VIII, XII and XIII were major clusters that contained between 5 and 28 strains. Clusters I, V, VI, VII, IX, X and XI were minor clusters with only 1–3 strains. Strains in cluster I grouped closely with the type strains of *B. yuanmingense*,*B. vignae*,*B. cajani*,*B. centrosematis* and/or *B. guangxiense*, respectively, and shared a sequence similarity of 96–100% with their respective reference strains. Strains in clusters II, III, IV, VII, VIII, IX, X, XII and XIII showed <96% sequence similarities with reference species and did not group with any described species. The phylogeny of 54 strains representing each *recA* cluster was further studied by the sequence analysis of *glnII* (423 bp),*gyrB* (419 bp),*rpoB* (435 bp),*atpD* (426 bp) and *dnaK* (246 bp). The phylogenies of the individual housekeeping genes are presented in Figs S1–S6 (Supporting Information). We reconstructed *recA* phylogeny with only 54 strains to compare the phylogenetic groups with other single gene phylogenies. The 13 clusters (I–XIII) obtained in Fig. 1 were also consistently recovered in the new *recA* phylogeny (Fig. S6, Supporting Information). The strains in the *gyrB* and *atpD* trees were grouped into 14 clusters each (Figs S1 and S2, Supporting Information). The *dnaK*, *rpoB* and *glnII* phylogenetic trees separated the strains into 9, 11 and 13 clusters, respectively (Figs S3–S5, Supporting Information). While strains (e.g. strains from *recA* clusters VI, VIII, IX, X, XI, XII, XIII) consistently clustered together across the single gene phylogenetic trees, other groups are also mostly consitent but showed aberrant clustering among strains and with reference strains only in one or two gene trees (e.g. strains from the *recA* clusters I, II, IV. V, VII) (Table 2; Figs S1–S6, Supporting Information).

### MLSA phylogeny

**Figure 2.**
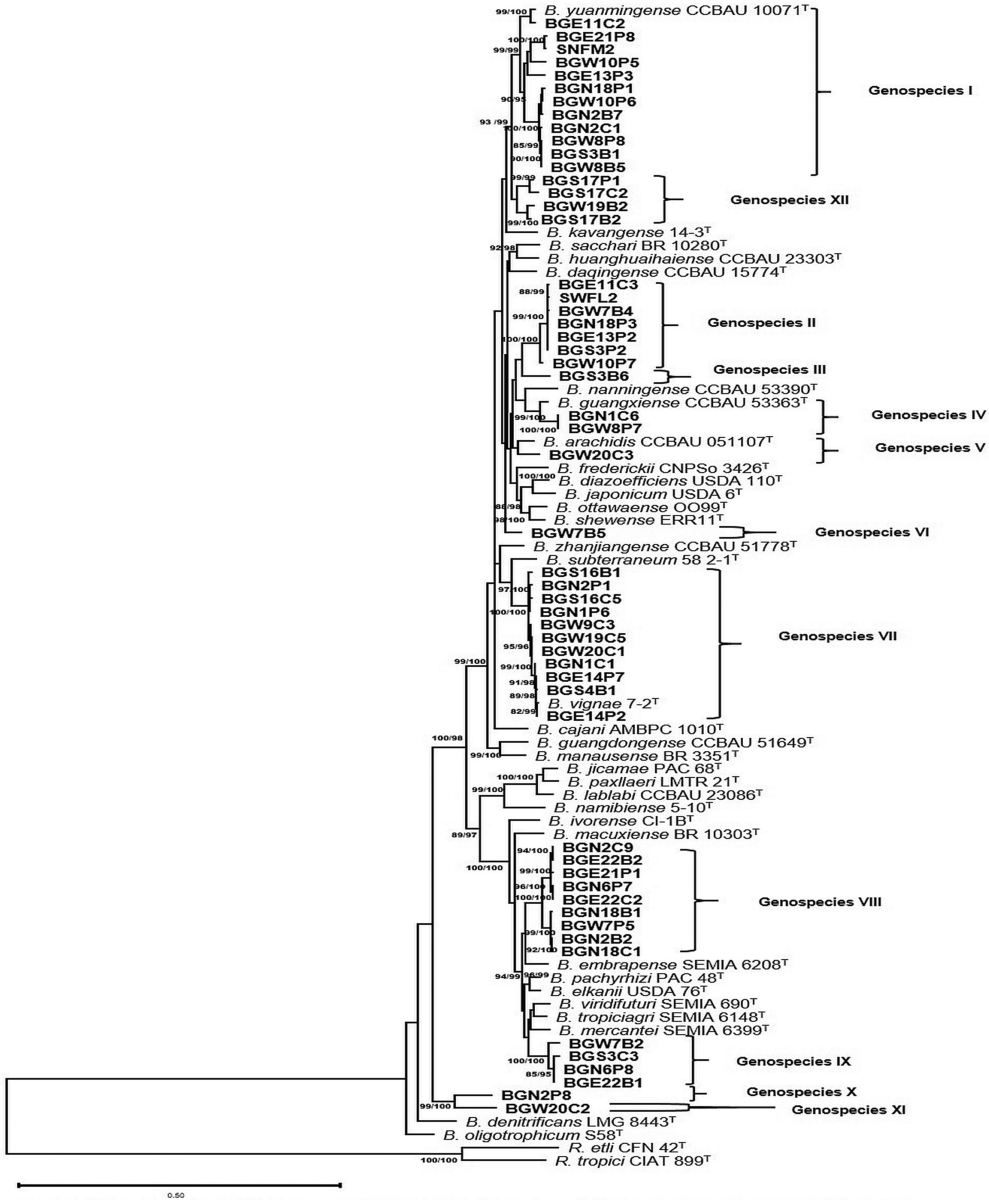
Rooted maximum likelihood phylogenetic tree based on *atpD-dnaK-glnII-gyrB-recA-rpoB* gene sequences showing relationship among the new isolates and reference strains in the genus *Bradyrhizobium*. SH-aLRT and ultrafast bootstrap values were inferred from 1000 replicates and are indicated at the nodes when SH-aLRT is ≥80% and ultrafast bootstrap is ≥95%. The scale bar indicates the estimated nucleotide substitution rate. *R. tropici* CIAT 899^T^ and *R. etli* CFN 42^T^ were used as outgroup.

To further refine the taxonomic position of the representative strains, a concatenated phylogenetic analysis based on six protein-coding housekeeping genes (*recA-glnII-gyrB-rpoB-dnaK-atpD*) was done. The concatenated sequence contained 2127 analyzed sites of which 1182 sites were conserved, 766 were distinct patterns and 783 were parsimony-informative (Table S2, Supporting Information). Considering phylogenetic groupings as an evolutionary parameter, the concatenated phylogeny confirmed the presence of diverse and novel type *Bradyrhizobium* species separated in twelve clusters designated as genospecies I–XII (Fig. 2). Most of the 54 representative strains clustered in the two well-known supergroups, *B. japonicum* (genospecies I–VII and XII) and *B. elkanii* (genospecies VIII and IX). The strains that formed genospecies X and XI however, clustered in a well-supported distinct branch (99/100). The information on the taxonomic affiliation of the genospecies is presented in Table 2. Genospecies I, II, VII, VIII, IX and XII were identified as major clusters containing 4–16 strains that were isolated from 2–10 locations. While four of the genospecies (I, IV, V and VII) identified in this study belonged to recognized *Bradyrhizobium* species, eight (II, III, VI, VIII, IX, X, XI and XII) were unique; forming separate clusters without including known close references. Some of the strains grouped under genospecies I formed aberrant clusters in the single gene phylogenies but were grouped with *B. yuanmingense* (Branch support (BS) = 99/99) in the concatenated phylogenetic tree. These strains were widely distributed in ten of the isolation sites of the study. The strains that constituted genospecies II did not cluster with reference strains in the concatenated phylogenetic tree. Strains BGS3B6, BGW7B5, BGN2P8 and BGW20C2 stood alone and did not cluster with known reference strains in the combined gene tree but showed distant relationship with *B. huanghuaihaiense* (95% sequence similarity), *B. arachidis* (95.9% sequence similarity) and *B. denitrificans* (91.3–92% sequence similarity), respectively. In genospecies IV, a close relationship was observed between *B. guangxiense* and strains BGN1C6 and BGW8P7 (BS = 99.8/100) that were isolated from two distinct locations in the Guinea savanna agroecological zone. The closest related species of genospecies V was *B. arachidis*, sharing a concatenated sequence similarity of 96.1%. Members of genospecies VII consistently clustered with *B. vignae* in all single gene trees and also in the concatenated phylogeny (BS = 100/100), sharing a sequence similarity range of 99.2–99.8% with the reference strain. The eleven strains included in genospecies VII were distributed across eight sampling sites (Table S3, Supporting Information). Genospecies VIII harbored only nine potentially unique strains from this study, sharing a sequence similarity of 95–95.3% in the concatenated phylogeny with the closest species *B. embrapense*. The cluster formed by strains in genospecies IX did not include known references and were far from the closest reference *B. mercantei* (95.4–95.9% sequence similarity). Genospecies XII was constituted by only four potentially unique strains identified in this study. These strains shared a sequence similarity range of 96.1–96.5% with the closest reference, *B. yuanmingense*. In general, while nine of the soils used in the study hosted both known and unnamed genospecies, four other soils harbored only strains identified as potentially unique species (Table S3, Supporting Information).

### Symbiotic phylogenies

**Figure 3.**
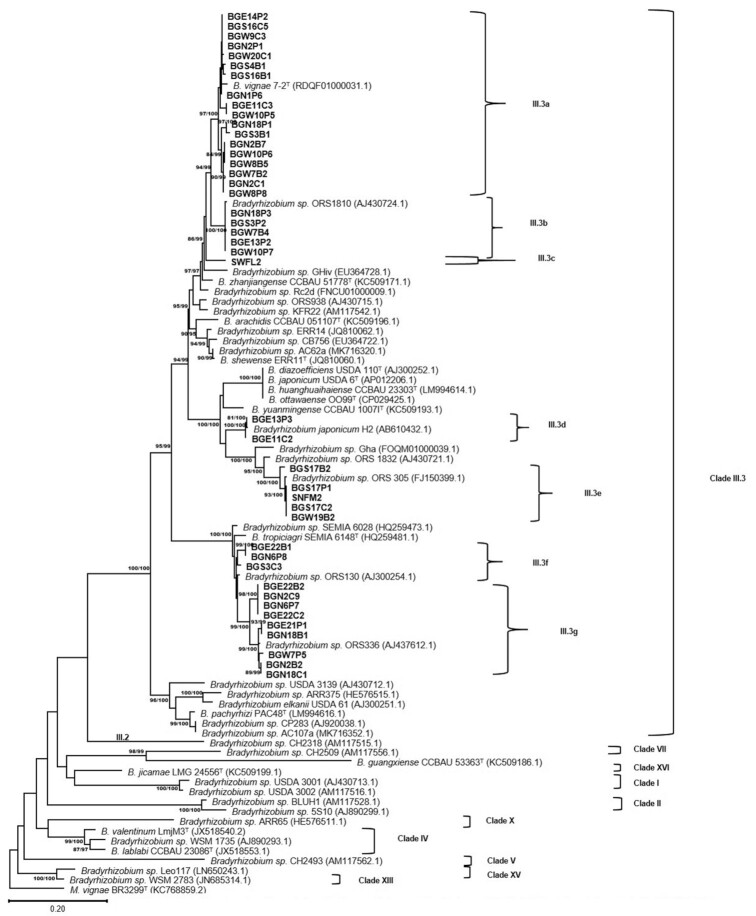
Maximum likelihood phylogeny based on *nodA* gene showing relationship among Bambara groundnut- and soybean-nodulating *Bradyrhizobium* test strains and references. The SH-aLRT and ultrafast bootstrap values were inferred from 1000 replicates and are indicated at the nodes when SH-aLRT is ≥80% and ultrafast bootstrap is ≥95%. The scale bar indicates the estimated nucleotide substitution rate. *M. vignae* BR3299^T^ was used as outgroup.

**Figure 4.**
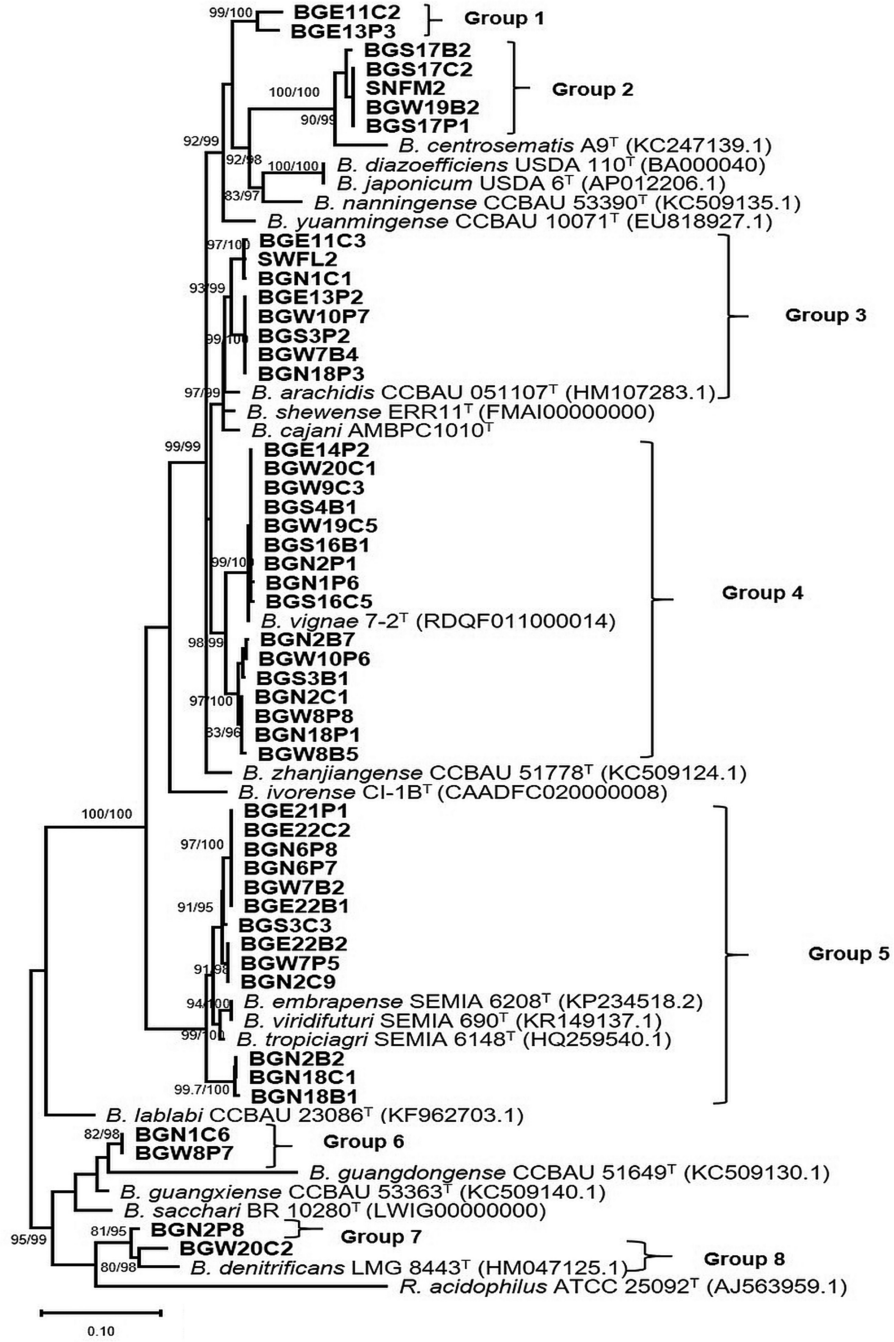
Maximum-likelihood phylogenetic tree based on sequences of *nifH* gene showing the relationship among new rhizobial isolates and recognized species in the *Bradyrhizobium* genus. SH-aLRT and ultrafast bootstrap values ≥80% and ≥95%, respectively (based on 1000 replications), are shown at each node. The scale bar indicates the estimated nucleotide substitution rate. *R. acidophilus* ATCC 25092^T^ was included as outgroup.

To determine the phylogenies of symbiotic genes, the nucleotide sequences of the *nodA* (465 bp) and *nifH* (559 bp) genes were obtained for 43 and 48 representative strains, respectively. The phylogenies of the *nodA* and *nifH* genes were examined by including relevant *Bradyrhizobium* reference sequences available from GenBank (Figs 3 and 4). Based on the *nodA* phylogenetic tree, all the representative strains were found to belong to *nodA* clade III.3 (Moulin *et al*. 2004, Stępkowski *et al*. 2005, 2012). The strains from this study were further delineated into seven subclusters within *nodA* Clade III.3. In Clade III.3a, 18 representative strains from four genospecies (I, II, VII and IX) grouped with the type strain of *B. vignae* (BS = 97/100) (Grönemeyer *et al*. 2016) originally isolated from *Vigna unguiculata* in Namibia. All strains from genospecies II (excluding strains SWLF2 and BGE11C3) clustered together in *nodA* Clade III.3b and grouped with *Bradyrhizobium*sp. ORS1810 (BS = 100/100) (Moulin *et al*. 2004) isolated from *Crotalaria lathyroides* in Senegal. Strain SWLF2, however, stood alone without clustering with any reference strain in Clade III.3c. The *nodA* Clade III.3d included *B. japonicum* strain H2 (BS = 81/100) (Risal *et al*. 2012) originally isolated from *Vigna radiata* in Nepal together with two *Bradyrhizobium* strains from genospecies I. Clade III.3e harbored five strains from two genospecies (I and XII) and clustered with the non-photosynthetic *Bradyrhizobium*sp. ORS305 (BS = 100/100) (Nzoué *et al*. 2009) isolated from *Aeschynomene schimperi* growing in Senegal. Clade III.3f was represented by three strains from Genospecies IX and grouped with *Bradyrhizobium*sp. ORS130 (Dupuy *et al*. 1994) originally isolated from *Faidherbia albida* growing in Senegal. The *nodA* Clade III.3g was occupied by nine strains from genospecies VIII and included *Bradyrhizobium*sp. ORS336 (BS = 99/100) (Molouba *et al*. 1999) isolated from *Aeschynomene afraspera* growing in Senegal.

The *nifH* of the representative strains were separated in seven distinct clusters identified as groups 1–8 (Fig. 4). Branches formed by the strains in the *nifH* groups 1–4 were mostly similar to those formed by *nodA* Clades III.3a, III.3b, III.3d and III.3e. Groups 1 and 2 did not cluster with any reference strain but in groups 3 and 4, the representative strains clustered closely with *B. arachidis* (BS = 93/99) and *B. vignae* (BS = 98/99), respectively. Strains within genospecies VIII and IX were separated into two clusters in the *nodA* (Clades III.3f and III.3g) phylogenetic tree but formed a single cluster in the *nifH* phylogeny (group 5). *nifH* groups 6, 7 and 8 did not include any reference strains and were represented by strains identified in this study as genospecies IV, X and XI, respectively. Generally, the *nodA* and *nifH* phylogenies of the representative strains were mostly not congruent with the concatenated phylogenetic tree.

### Effect of soil physicochemical factors on the distribution of genospecies and *recA* groups

**Figure 5.**
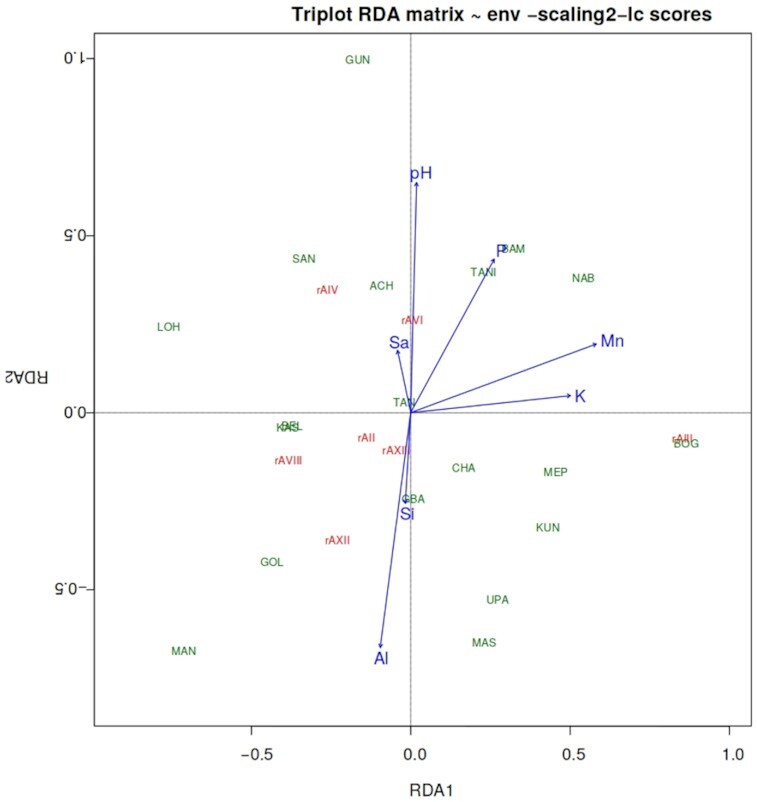
Biplot of the RDA on *recA* (rA) clusters and their soil factors from sampling sites in the northern savanna zones of Ghana. Si, silt; P, phosphorus; Sa, sand; Al, exchangeable acidity; K, potassium; Mn, manganese. Short names are the names of the sampling sites.

**Figure 6.**
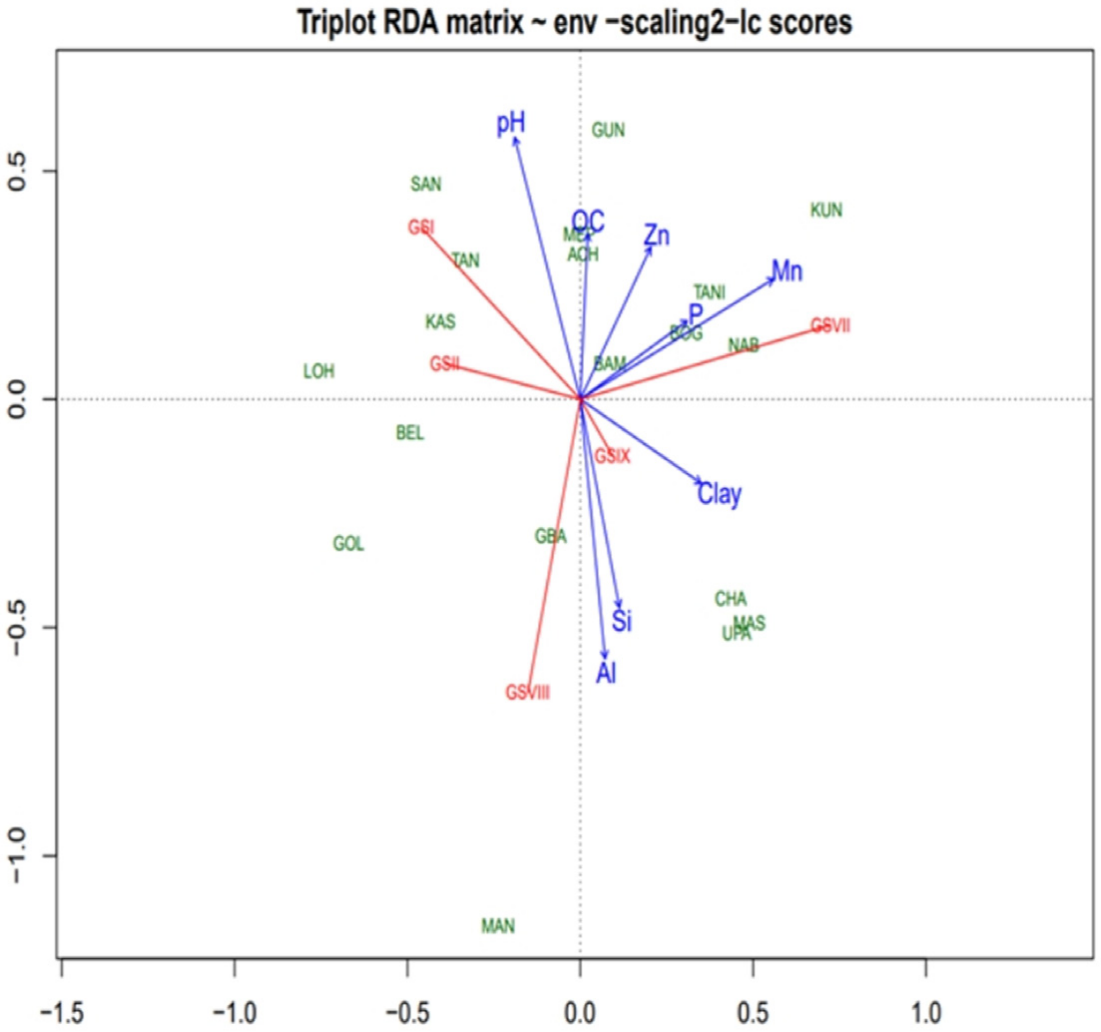
Biplot of the RDA on genospecies (GS) and their soil factors from sampling sites in the northern savanna zones of Ghana. Al, exchangeable acidity; Si, silt; Mn, manganese; P, phosphorus; Zn, zinc; OC, organic carbon. Short names are the names of the sampling sites.

The MANOVA test result revealed that the soil variables were significantly different (*P* < 0.001, data not shown) among *recA* clusters, and between the genospecies (Table 1; Table S3, Supporting Information). In the RDA result presented in Fig. 5, the first two axes explained most of the variation (cumulative 40%) presented between the *recA* clusters across the sampling sites and thus, the two axes in the plot represented the data well. The soil pH, exchangeable acidity (Al), Mn, K, and among the textural fractions sand (Sa) and silt (Si) appeared to be the main factors affecting the distribution of the *recA* clusters. The distribution of *recA* clusters rAIV and rAVI tended to follow the soil pH and sand contents. Soil exchangeable Al and Si exhibited a positive influence on the distribution of *recA* clusters rAVIII, rAXII and rAXIII. The soil Mn and K showed to have a positive effect on the distribution of rAII, but negatively affected the distribution of rAII, rAVIII and rAXIII. Soil P also had a negative effect on the distribution of rAII, rAVIII, rAXII and rAXIII. As shown in Fig. 6, in the RDA plot the first two axes explained 45% of the variations present between the genospecies across sampling sites. The soil pH, Mn and Al appeared to have the strongest effect on the distribution of the genospecies, followed by the silt fraction of the soil and the soil P content. The distribution of GSI and GSII appeared to be influenced positively by soil pH and negatively by clay content. The soil Mn and Al had a positive effect on distribution of the test strains that formed GSVII and GSVIII, respectively. Although the clay and Si tended to have an influence on the distribution of GSIX, the genospecies held a position closer to the center of the plot, suggesting that the contribution of the soil variables to the distribution of this genospecies was small.

## Discussion

The BNF process contributes greatly to the environment and to agricultural sustainability. Its success during the introduction of new legume crops largely depends on the selection of highly effective rhizobial strains, preferable from the native population. These populations, however, contain diverse rhizobial species with varied effectiveness. Thus, an understanding of the diversity and taxonomy of native rhizobial species is a crucial step toward improving legume productivity using BNF. We analyzed rhizobial isolates recovered from root nodules of Bambara groundnut and soybean grown in soils sampled from the northern savanna zones of Ghana to determine their diversity and taxonomic affiliation. The northern savanna zones are characterized by harsh climates and heterogeneous soils that are generally low in plant nutrients (Bationo *et al*. 2018). Our sampling sites had no history of inoculation and therefore the rhizobia recovered in this study represent an indigenous population that is well adapted to the local environmental conditions.

By combining the phylogenetic information of several protein-coding housekeeping genes, MLSA allows adequate classification of closely related rhizobial species (Mousavi *et al*. 2015, Grönemeyer *et al*. 2016, Chidebe *et al*. 2018). This reduces the possible impact of horizontal gene transfers (HGTs) that could affect single gene analysis (Gevers *et al*. 2005, Martens *et al*. 2008). At least four to five genes should be considered for MLSA (Gevers *et al*. 2005). In our study, we used six housekeeping genes (*recA*,*glnII*,*dnaK*,*atpD*,*gyrB* and *rpoB*) to generate phylogenetic trees and subsequently establish phylogenies. Considering mainly the phylogenetic groupings of the strains,, our MLSA results revealed the presence of diverse groups of *Bradyrhizobium* as predominant symbionts of Bambara groundnut and soybean. According to Martínez-Romero and Caballero-Mellado (1996), rhizobial diversity is higher in centers of host plant origins than in environments where the host has been introduced. Correspondingly, Andronov *et al*. (2003) and Österman *et al*. (2011) reported that the diversity of *Neorhizobium galegae* sv. orientalis is higher in the Caucasus that is the center of origin for its host, *Galega orientalis*, than the diversity of *N. galegae* sv. officinalis is in this area. Our study revealed diverse indigenous *Bradyrhizobium* species as nodule symbionts of Bambara groundnut in Africa, the center of origin for this legume (Grönemeyer *et al*. 2014, Puozaa *et al*. 2017, Ibny *et al*. 2019). Also, its distinct and diverse genospecies identified as symbionts confirm the promiscuity of this legume (Grönemeyer *et al*. 2014, Puozaa *et al*. 2017, Ibny *et al*. 2019).

According to the phylogenetic analysis of the concatenated sequences, many of the representative *Bradyrhizobium* strains were classified into two main lineages, the *B. japonicum* and *B. elkanii* supergroup. The *B. japonicum* supergroup is reported to have a much broader geographical distribution than the *B. elkanii* supergroup (Vinuesa *et al*. 2008, Stępkowski *et al*. 2018), and many of the strains known to be nodule symbionts of the diverse leguminous crops cultivated in Africa are affiliated to species of this supergroup (Jaiswal and Dakora 2019). For instance, *B. kavangense* (Grönemeyer *et al*. 2015a) and *B. daquingense* (Steenkamp *et al*. 2008) were identified as microsymbionts of cowpea in soils of Southern Africa (Angola and Namibia) and Botswana, respectively. In Namibian and Ghanaian soils, respectively, strains of *B. subterraneum* (Grönemeyer *et al*. 2015b) and *B. vignae* (Puozaa *et al*. 2017) have been isolated from nodules of Bambara groundnut. Likewise, *B. japonicum* and *B. yuamingense* have been found associating with soybean and groundnut, respectively, in Mozambique (Chibeba *et al*. 2017) and Ghana (Osei *et al*. 2018), and many of the strains known to be nodule symbionts of the diverse leguminous crops cultivated in Africa are affiliated to species of this supergroup (Jaiswal and Dakora 2019). For instance, *B. kavangense* (Grönemeyer *et al*. 2015a) and *B. daquingense* (Steenkamp *et al*. 2008) were identified as microsymbionts of cowpea in soils of Southern Africa (Angola and Namibia) and Botswana, respectively. In Namibian and Ghanaian soils, respectively, strains of *B. subterraneum* (Grönemeyer *et al*. 2015b) and *B. vignae* (Puozaa *et al*. 2017) have been isolated from nodules of Bambara groundnut. Likewise, *B. japonicum* and *B. yuamingense* have been found associating with soybean and groundnut, respectively, in Mozambique and Ghana. In our study also the members that constituted the *B. japonicum* lineage were found in nearly 90% of the isolation sites, relative to the *B. elkanii* supergroup that was only limited to seven sampling sites.

Of the twelve genospecies we identified, four of the genospecies were conspecific with recognized *Bradyrhizobium* species in the *B. japonicum* supergroup. The remaining strains (except BGN2P8 and BGW20C2) that were distributed in both the *B. japonicum* and *B. elkanii* supergroups, appeared unique and separated from all described species. These unique genospecies could most likely be novel *Bradyrhizobium* species whose identities can be justified through further taxonomic analysis and by applying a set of criteria designed for bacterial species description (De Lajudie *et al*. 2019). Also, strains of the potentially unique groups were widespread in soils of twelve locations, with the soils of four locations only containing strains of the unique groups identified in this study. Our results corroborate various diversity studies conducted in Africa that have shown that novel *Bradyrhizobium* species are dominant and widespread in many African soils (Steenkamp *et al*. 2008, Aserse *et al*. 2012b, Grönemeyer *et al*. 2014, Puozaa *et al*. 2017, Mohammed *et al*. 2018). Thus, this calls for continuous exploration of new geographical areas to uncover the full diversity that may provide a vast resource for development of adapted inoculants that can greatly profit regions where smallholder farming is widespread. The species *B. pachyrhizi*,*B. vignae*,*B. yuanmingense*,*B. daqingense* and *B. subterraneum* have been reported as nodule symbionts of Bambara groundnut in African soils (Grönemeyer *et al*. 2015b, 2014, Puozaa *et al*. 2017, Ibny *et al*. 2019). In this study also, strains from genospecies I and VII were related to *B. yuanmingense* and *B. vignae*, respectively. *Bradyrhizobium yuanmingense*, originally recovered from *Lespedeza cuneata*, a native tree grown in China (Yao *et al*. 2002) has a broad distribution and host range. This species has been isolated from diverse geographical regions, e.g. Mexico (Vinuesa *et al*. 2008), Botswana (Steenkamp *et al*. 2008), Peru (Ormeno-Orrillo *et al*. 2006), Ghana (Osei *et al*. 2018), ) and Mozambique (Chidebe *et al*. 2018) and from nodules of legumes such as cowpea, groundnut and lima bean. *Bradyrhizobium vignae* however, is mainly distributed in Africa from countries such as Senegal (Li *et al*. 2015), Namibia (Grönemeyer *et al*. 2016), South Africa and Ghana (Puozaa *et al*. 2017). In our study, genospecies IV and V had a close relationship with *B. guangxiense* and *B. arachidis*, respectively, both originally isolated from *Arachis hypogaea* in China (Wang *et al*. 2013b, Li *et al*. 2015). To the best of our knowledge, our study is the first to have isolated strains of *B. guangxiense* and this recognition expands knowledge on the known symbionts of Bambara groundnut.

Physical and chemical properties of soil play an important role in influencing microbial communities, composition and functions (Zhang *et al*. 2011). Edaphic parameters such as pH, organic carbon (C), particle size distribution, nitrogen (N), calcium (Ca) and magnesium (Mg) have been shown to be the main determinants of legume rhizobial distribution in many geographical locations (Puozaa *et al*. 2019, Asfaw *et al*. 2020). There are reports of geographical locations with soils predominantly composed of clay and silt showing higher rhizobial diversity than in locations with larger soil particle sizes (Sessitsch *et al*. 2002). Other studies have also shown pH as a major determinant of bacterial diversity and distribution (Giongo *et al*. 2008, Puozaa *et al*. 2017). In the current study, generally, the soil properties of the sampling sites could have influence the distribution of both the *recA* clusters and genospecies. The soil pH, clay, silt and sand content, and exchangeable Al, Mn and K were among the main factors influencing the distribution of the majority of *recA* clusters and/or the genospecies (Figs 5 and 6). It should be emphasized that the concentration of exchangeable Al reflects soil pH and this study thus also shows the importance of soil acidity in the composition of soil microbiota (Ndungu *et al*. 2018, Puozaa *et al*. 2019).

The *nodA* and *nifH* genes are important symbiotic genes that code for nodulation and nitrogen fixation, respectively. Usually, these genes are located on symbiotic islands that most likely increases the possibility of gene transfer between different bacterial species (Haukka *et al*. 1998, Andrews *et al*. 2018). As such, the observation that some of our nodule isolates could not reinfect their homologous host may have been as a result of a loss of the symbiotic loci as was also observed in a study conducted by (Angelini *et al*. 2011). Furthermore, rather than showing species relationship, in our study the *nodA* gene appears to reveal information on geographic origin or host range. Studies by several authors have demonstrated the monophyletic origin of this gene in *Bradyrhizobium* (Moulin *et al*. 2004, Stepkowski *et al*. 2007, Aserse *et al*. 2012b, Beukes *et al*. 2016). Recently, 16 *nodA* clades designated as Clades I–XVI have been described for this genus, with the Clade III being highly diversified and cosmopolitan, including members that are widespread in Sub-Saharan Africa (SSA), Australia, the Americas and in southern and eastern Asia (Stępkowski *et al*. 2007, Steenkamp *et al*. 2008, Beukes *et al*. 2016). The majority of *Bradyrhizobium* strains isolated from SSA form part of *nodA* clade III and are able to establish symbiosis with both native and cultivated legumes (Steenkamp *et al*. 2008, Asers *et al*. 2012b, Degefu *et al*. 2017, 2018). Accordingly, all strains detected in this study belonged to the *nodA* Clade III and were clustered with reference species mainly originating from Africa. It has been widely reported that the phylogenies of the symbiotic and housekeeping genes often show different evolutionary histories due to HGT (Moulin *et al*. 2004, Stępkowski *et al*. 2005, Menna and Hungria 2011).

The events of HGT result in phylogenetically distant microsymbionts carrying similar symbiotic genes (Horn *et al*. 2014). Our results also showed that the *nodA* and *nifH* phylogenies were largely incongruent with the combined housekeeping gene tree. For instance, strains from genospecies I were divided into three groups under the highly diverse *nodA* Clade III.3 (assigned as III.3a, III.3b and III.3d). Similarly, genospecies II and IX were distributed in *nodA* Clades III.3c–e and III.3d–f, respectively. Also, the representative test strains from genospecies VIII and IX formed a single *nifH* group (group 5). These results are consistent with findings of other authors and show that HGT is a common phenomenon in symbiotic genes of the genus *Bradyrhizobium* (Moulin *et al*. 2004, Koppell and Parker 2012, Huang *et al*. 2018). Notwithstanding these observations, the consistent clustering of *B. vignae* with some of our strains in both symbiotic and individual, as well as combined housekeeping gene phylogenies, reflects a possible vertical gene transfer that was also reported in previous studies (Aserse *et al*. 2012b, Chen *et al*. 2014). Furthermore, the results showed representative strains in *nifH* groups 1, 2 and 6 did not group closely with known reference strains that could indicate the presence of unique *nifH* variants for Bambara groundnut and soybean rhizobia in Ghana.

## Conclusion

From the results obtained in this study, we conclude that the Bambara groundnut is nodulated by diverse and unique *Bradyrhizobium* genospecies that appear to be widely distributed in the soils of the northern savanna zones of Ghana. The unnamed genospecies, which potentially represent novel species, would require further characterization for valid description. The phylogenies of the housekeeping genes were generally incongruent with the symbiotic genes indicating an independent evolutionary history between the housekeeping genes and the symbiotic genes. The soil factors analyzed in the study contributed to structuring the bradyrhizobial communities detected at the sampling sites.

## Acknowledgments

The authors express their appreciation to Petri Penttinen of the Faculty of Biological and Environmental Sciences, ECOENV, for proofreading this document. We acknowledge the invaluable support from Professor John Sumelius of the Department of Economics and Management, University of Helsinki. We are grateful to the University of Helsinki, Faculty of Biological and Environmental Sciences and the Council for Scientific and Industrial Research–Savanna Agricultural Research Institute for the technical and infrastructural assistance.

## Supplementary Material

fiac043_Supplemental_Files
