# Supplementary material for: Phylogenetically diverse Bradyrhizobium genospecies nodulate Bambara groundnut (Vigna subterranea L. Verdc) and soybean (Glycine max L. Merril) in the northern savanna zones of Ghana
Source: FEMS Microbiol Ecol. 2022 Apr 11;98(5):fiac043. doi: 10.1093/femsec/fiac043 (PMC9329091; doi:10.1093/femsec/fiac043)
Supplement: fiac043_Supplemental_Files [file fiac043_supplemental_files.zip › Revised_Supplementary_Data_figures.docx]

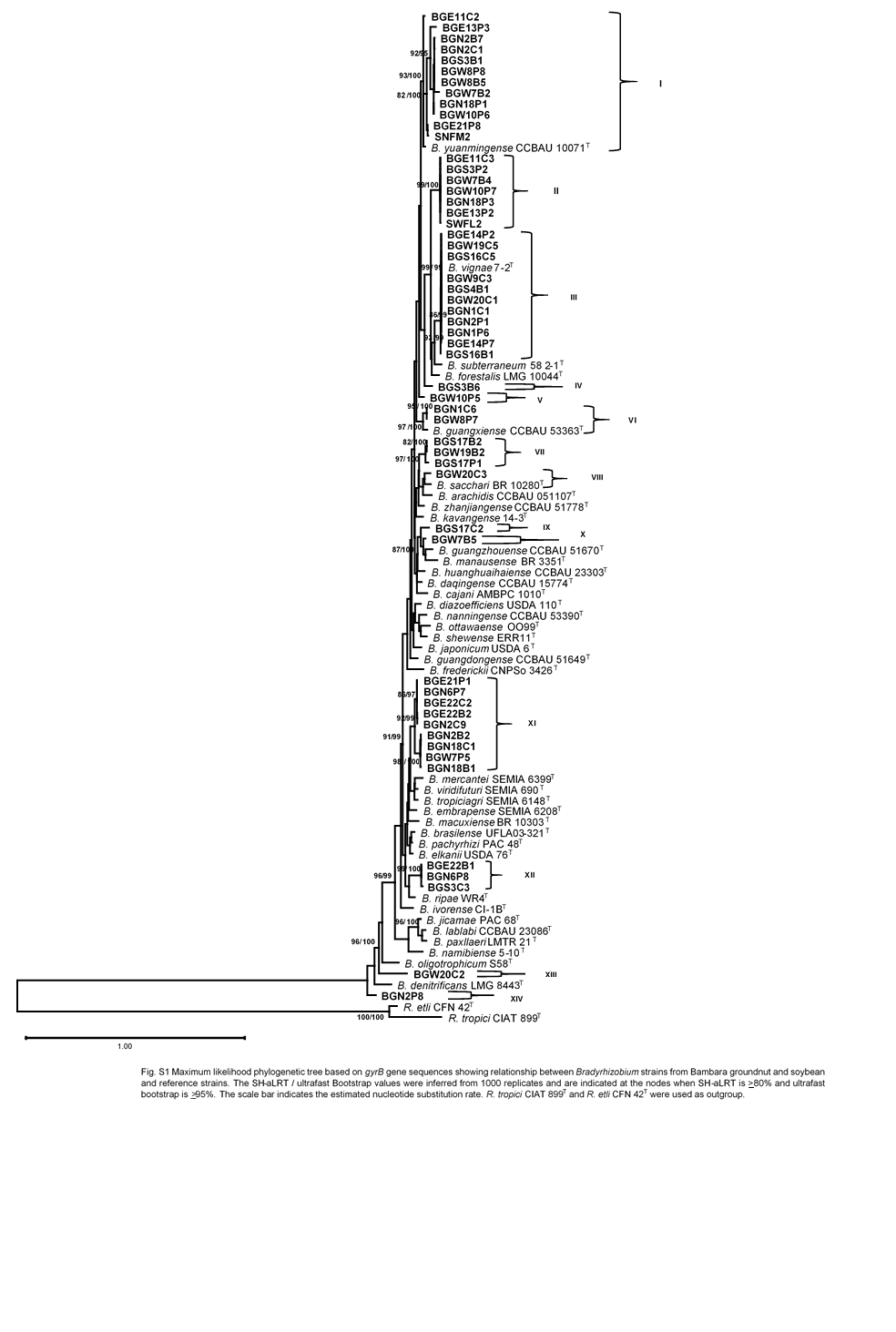


Figure S1. Maximum likelihood phylogenetic tree based on *gyrB* gene sequences showing relationship between *Bradyrhizobium* strains from Bambara groundnut and soybean and reference strains. The SH-aLRT / ultrafast Bootstrap values were inferred from 1000 replicates and are indicated at the nodes when SH-aLRT is >80% and ultrafast bootstrap is >95%. The scale bar indicates the estimated nucleotide substitution rate. *R. tropici* CIAT 899T and *R. etli* CFN 42T were used as outgroup.


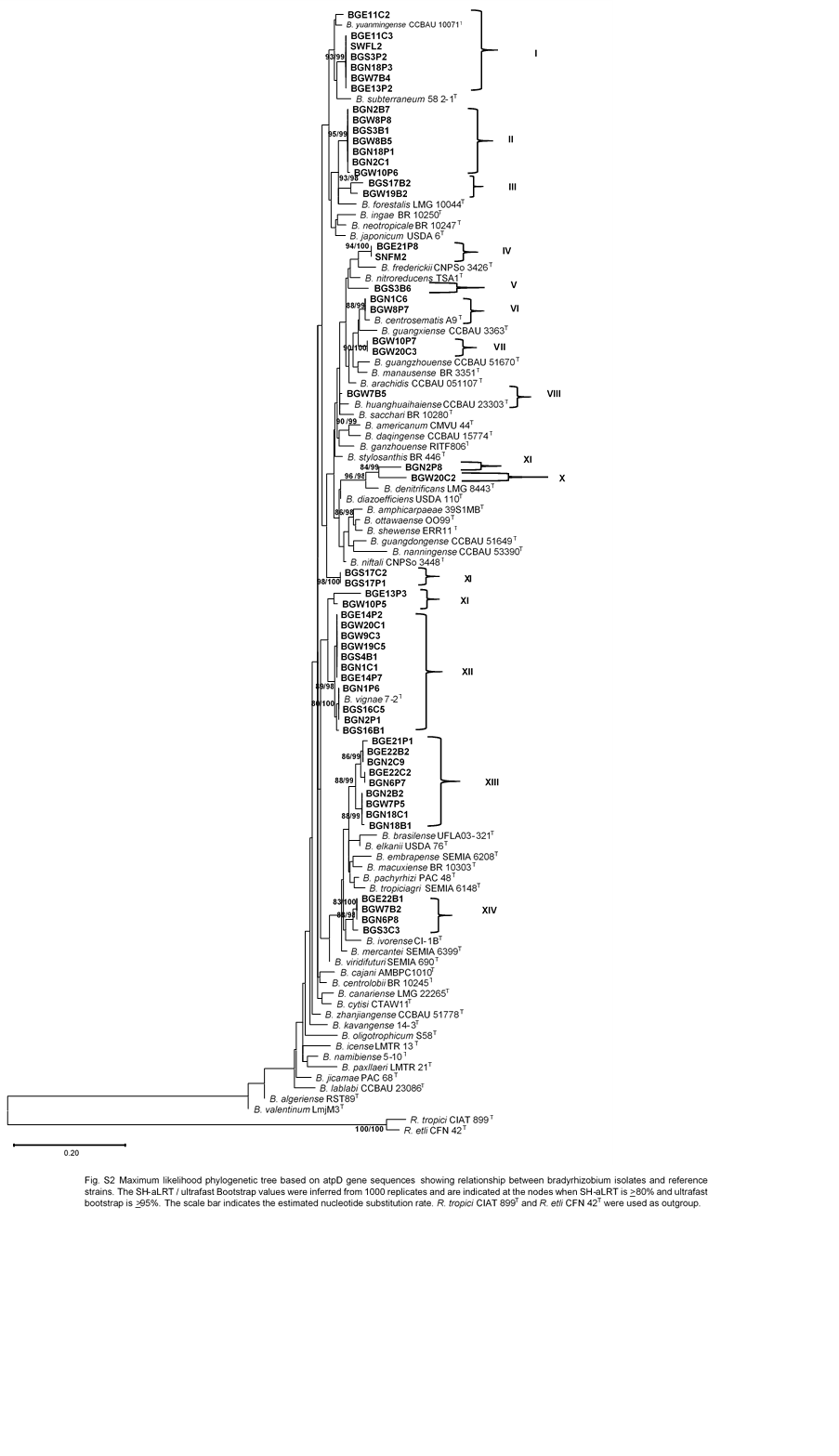


Figure S2. Maximum likelihood phylogenetic tree based on *atpD* gene sequences showing relationship between *Bradyrhizobium* strains from Bambara groundnut and soybean and reference strains. The SH-aLRT / ultrafast Bootstrap values were inferred from 1000 replicates and are indicated at the nodes when SH-aLRT is >80% and ultrafast bootstrap is >95%. The scale bar indicates the estimated nucleotide substitution rate. *R. tropici* CIAT 899^T^ and *R. etli* CFN 42^T^ were used as outgroup.


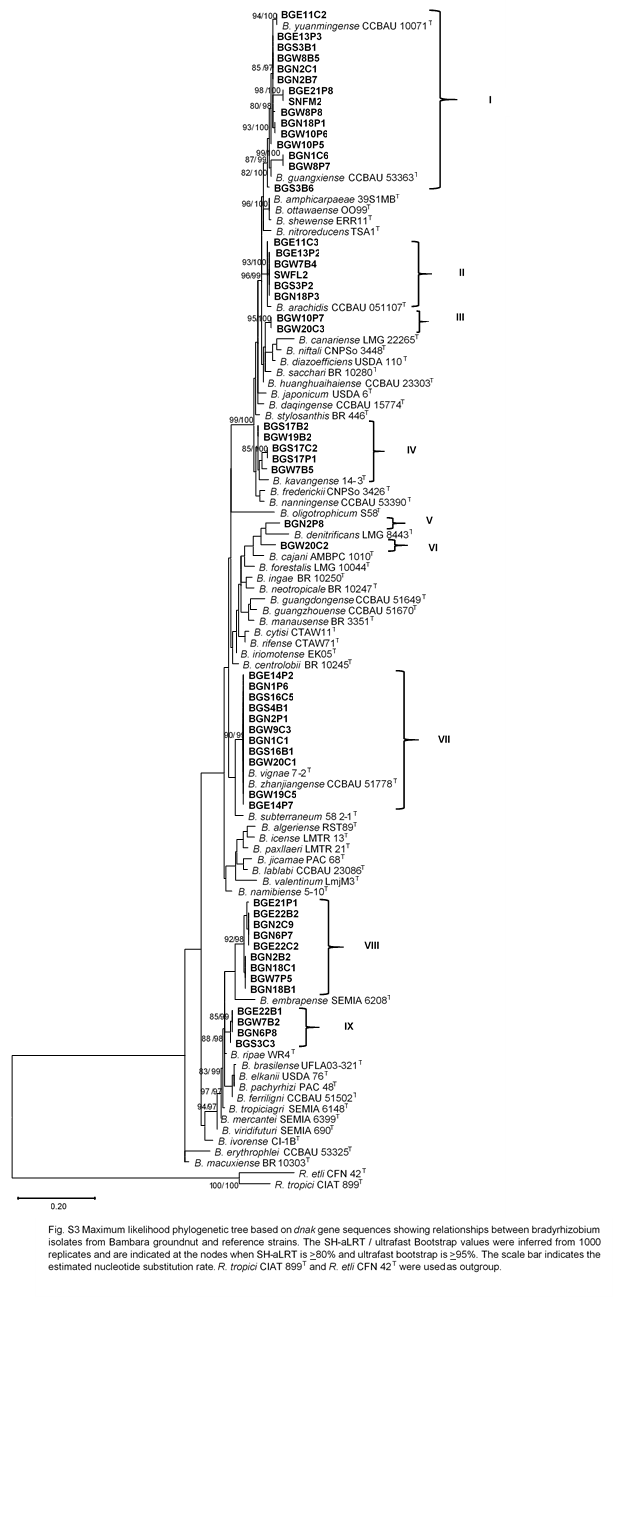


Figure S3. Maximum likelihood phylogenetic tree based on *dnak* gene sequences showing relationships between *Bradyrhizobium* strains from Bambara groundnut and soybean and reference strains. The SH-aLRT and ultrafast bootstrap values were inferred from 1000 replicates and are indicated at the nodes when SH-aLRT is >80% and ultrafast bootstrap is >95%. The scale bar indicates the estimated nucleotide substitution rate. *R. tropici* CIAT 899^T^ and *R. etli* CFN 42^T^ were used as outgroup.


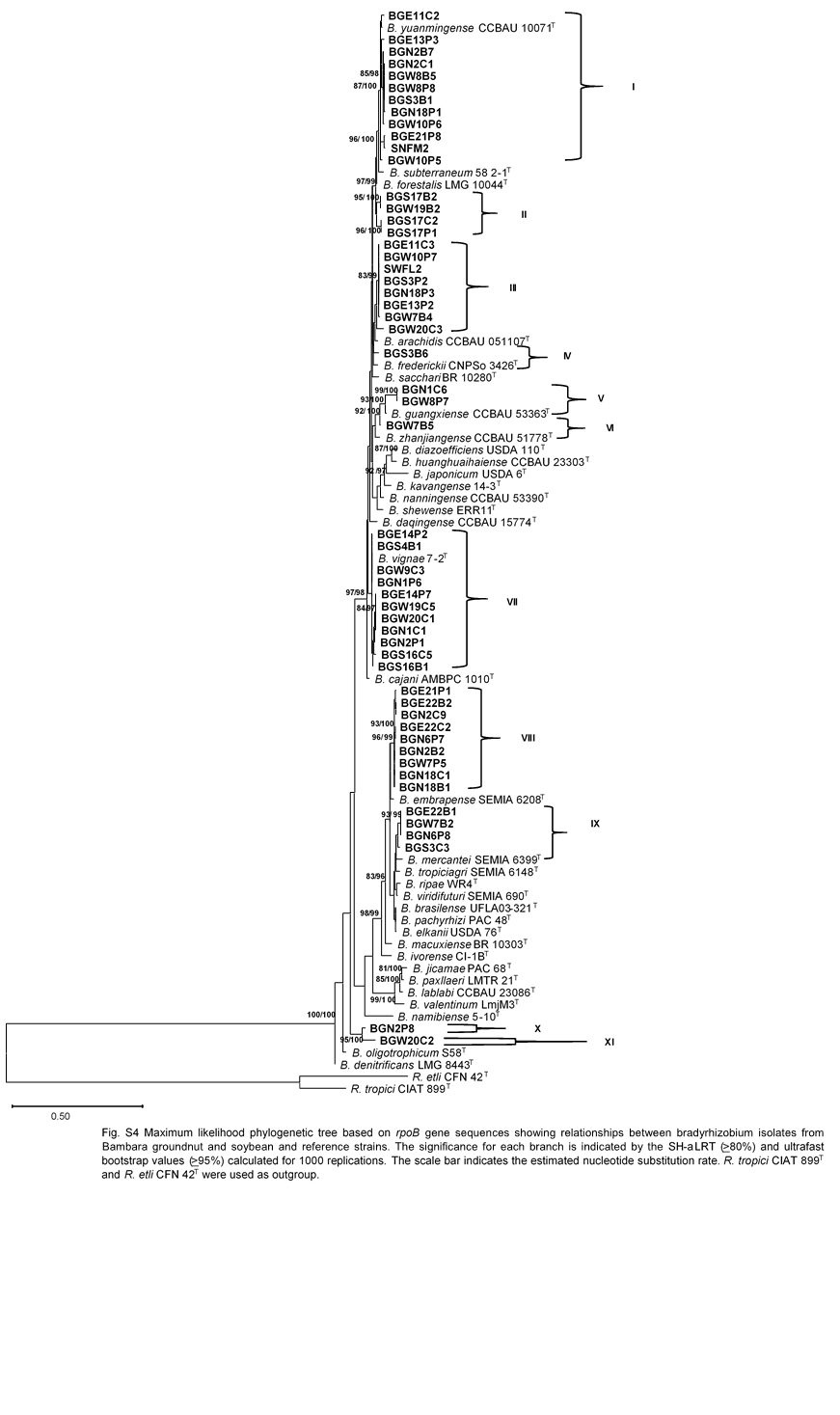


Figure S4. Maximum likelihood phylogenetic tree based on *rpoB* gene sequences showing relationships between *Bradyrhizobium* strains from Bambara groundnut and soybean and reference strains. The significance for each branch is indicated by the SH-aLRT (>80%) and ultrafast bootstrap values (>95%) calculated for 1000 replications. The scale bar indicates the estimated nucleotide substitution rate. *R. tropici* CIAT 899^T^ and *R. etli* CFN 42^T^ were used as outgroup.


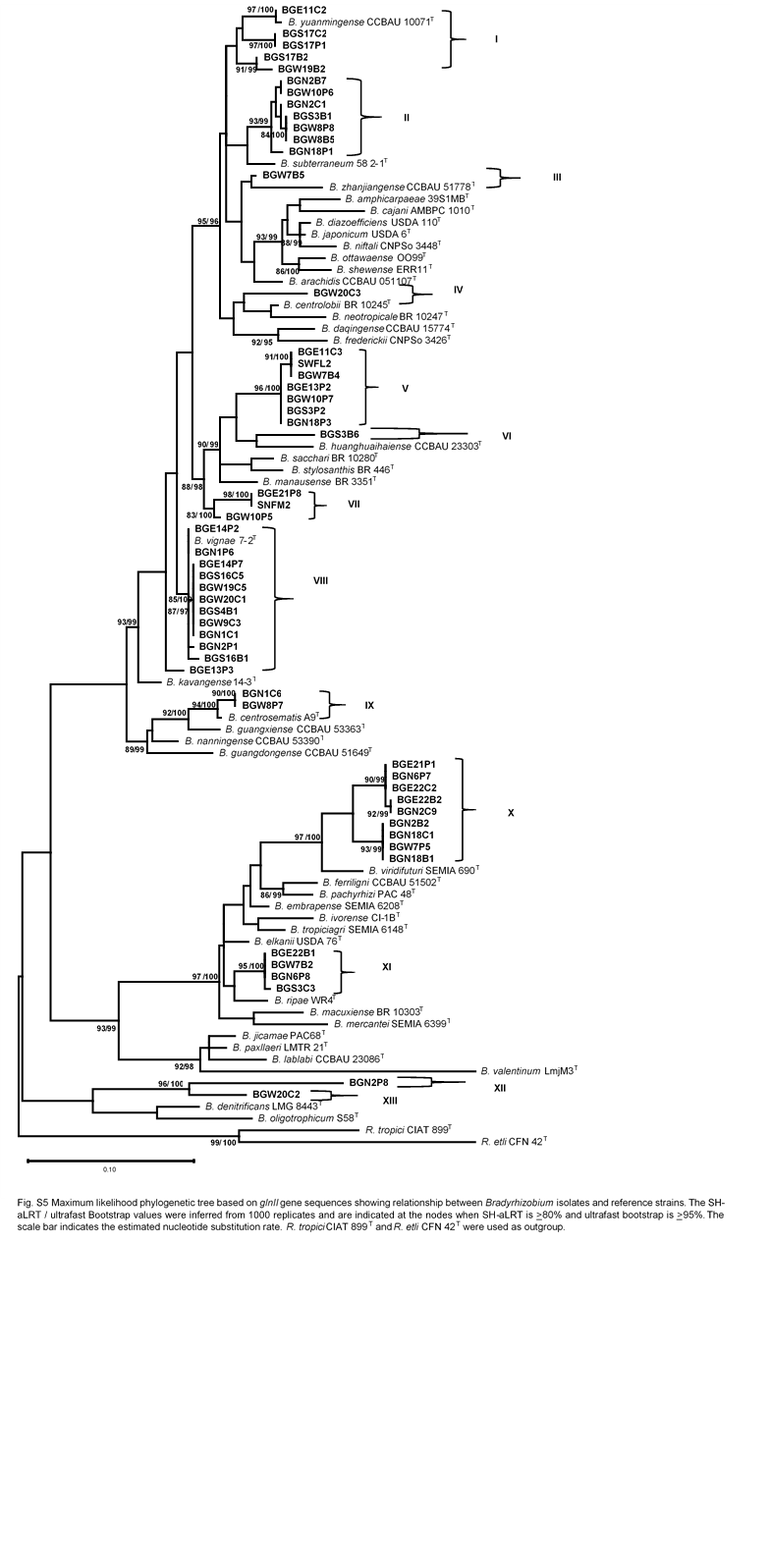


Figure S5. Maximum likelihood phylogenetic tree based on *glnII* gene sequences showing relationship between *Bradyrhizobium* strains from Bambara groundnut and soybean and reference strains. The SH-aLRT and ultrafast Bootstrap values were inferred from 1000 replicates and are indicated at the nodes when SH-aLRT is >80% and ultrafast bootstrap is >95%. The scale bar indicates the estimated nucleotide substitution rate. *R. tropici* CIAT 899^T^ and *R. etli* CFN 42^T^ were used as outgroup.


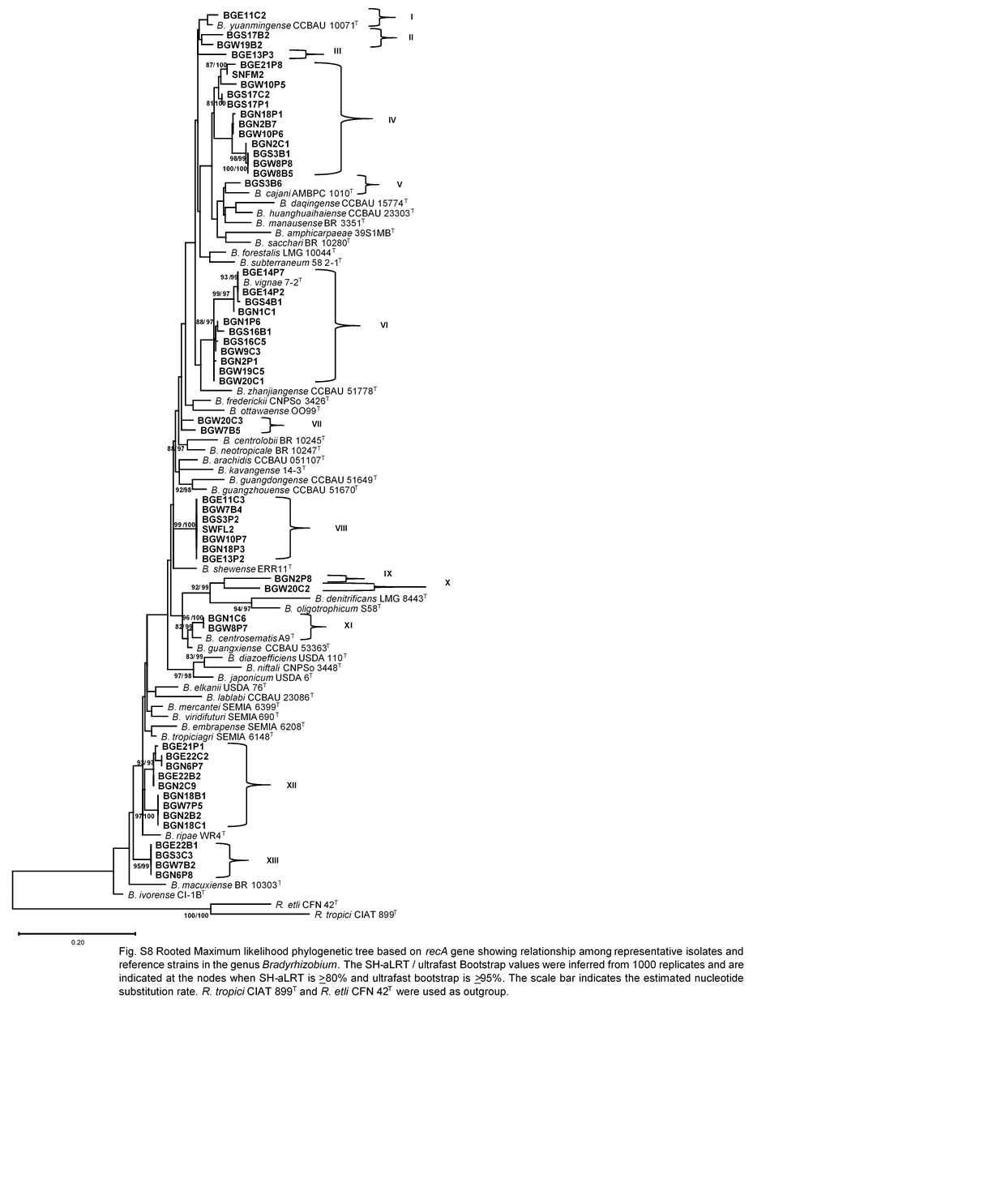


Figure S6. Rooted Maximum likelihood phylogenetic tree based on *recA* gene showing relationship among representative 54 *Bradyrhizobium* strains from Bambara groundnut and soybean and reference strains in the genus *Bradyrhizobium*. The SH-aLRT / ultrafast Bootstrap values were inferred from 1000 replicates and are indicated at the nodes when SH-aLRT is >80% and ultrafast bootstrap is >95%. The scale bar indicates the estimated nucleotide substitution rate. *R. tropici* CIAT 899^T^ and *R. etli* CFN 42^T^ were used as outgroup.
