# Supplementary material for: Phylogenetically diverse Bradyrhizobium genospecies nodulate Bambara groundnut (Vigna subterranea L. Verdc) and soybean (Glycine max L. Merril) in the northern savanna zones of Ghana
Source: FEMS Microbiol Ecol. 2022 Apr 11;98(5):fiac043. doi: 10.1093/femsec/fiac043 (PMC9329091; doi:10.1093/femsec/fiac043)
Supplement: fiac043_Supplemental_Files [file fiac043_supplemental_files.zip › Revised_supplementary_data_tables.docx]

**Table S1_** **supplementary data. Distribution of isolates in sampling sites.**

| **REGION** | **LOCATION** | **COORDINATES** | **SOIL TYPE** | **STRAIN** |
| --- | --- | --- | --- | --- |
| **UPPER EAST** | TANSIA | N 10° 56´ 27.8"  W 000° 19´ 23.9" | LIXISOL | BGE11C1, BGE11C2,  BGE11C3, BGE11C4 |
|  | KASIESA | N 10° 29 ´ 47.7"  W 001° 18´ 14.8" | LEPTOSOL | BGE13P2, BGE13P3,  BGE13P8 |
|  | BOGRIGO | N 10° 54´ 44.3''  W 000° 47´ 66.8'' | LIXISOL | BGE14P2, BGE14P7 |
|  | BELEMPIISI | N 10° 52´ 08.7"  W 000° 15´ 12.4" | GLEYSOL | BGE21P1, BGE21P4,  BGE21P8, BGE21B8,BGE21B9 |
|  | MANGA | N 11° 00´ 92.1"  W 000° 15´ 78.2" | ACRISOL | BGE22B1, BGE22B2,  BGE22C1, BGE22C2,  BGE22C4 |
| **NORTHERN** | GOLINGA | N 09° 20' 59"  W 000° 56' 40" | ACRISOL | BGN18B1, BGN18B2, BGN18B3,  BGN18B6, BGN18C1, BGN18P3,  BGN18C8, BGN18P1, BGN18P3,BGN18P5 |
|  | UPANDO | N 08 29´ 29.7"  W 000° 31´42.8" | PLINTHOSOL | BGN1C1, BGN1C2, BGN1C3, BGN1C4,  BGN1C6, BGN1C7, BGN1P9, BGN1P1,  BGN1P6 |
|  | MASAKA | N 08 38´ 37.4"  W 000° 31' 45.1'' | PLINTHOSOL | BGN2B2, BGN2B7, BGN2C1 BGN2C9,  BGN2P1, BGN2P6,BGN2P7, BGN2P8 BGN2P9, SNFM1,SNFM2, SNFM3, SNFM4 |
|  | CHANGNAAYILI | N 11° 00´ 9.2"  W 000° 15´ 7.8" | ACRISOL | BGN6P7, BGN6P8, BGN6P9 |
| **SAVANNA** | MEPASEM | N 09° 08´55.4"  W 001° 36´47.4" | LIXISOL | BGS16B1, BGS16B2, BGS16B3, BGS16C5 |
|  | GUNAYILI | N 10° 03´ 42.9"  W 000° 26´14.5" | LIXISOL | BGS17B1, BGS17B2, BGS17C2, BGS17P1 |
|  | ACHUBONYOR | N 09° 07´18.0"  W 001° 40´ 05.4" | LIXISOL | BGS3B1, BGS3B5, BGS3B6, BGS3B9,  BGS3C3, BGS3C4, BGS3C6, BGS3P2,  BGS3P7 |
|  | NABORI | N 09° 08´ 35.8"  W 000° 50´ 56.5" | PLANOSOL | BGS4B1 |
| **UPPERWEST** | LOHO | N 10° 8´ 41"  W 2° 32´ 7" | LIXISOL | BGW10P2, BGW10P4, BGW10P5,  BGW10P6, BGW10P7, SWFL2,  SWFL4, SWFL5 |
|  | TANINA | N 9° 53´ 11"  W 2° 27´ 49" | LIXISOL | BGW19B2, BGW19C4, BGW19C5 |
|  | BAMAHU | N 9° 59´ 30"  W 2° 28´ 8" | LIXISOL | BGW20C1, BGW20C2, BGW20C3 BGW20P1 |
|  | GBANKO | N 10° 12´ 35"  W 2° 32´ 55" | VERTISOL | BGW7B1, BGW7B2, BGW7B3, BGW7B4,  BGW7B5, BGW7B6, BGW7P5 |
|  | SANWANA | N 10° 11´ 45"  W 2° 34´ 10.55" | VERTISOL | BGW8B5, BGW8P7, BGW8P8 |
|  | KUNFABIALA | N 9° 59´ 5"  W 2° 28´ 2" | LEPTOSOL | BGW9B1, BGW9B3, BGW9B4, BGW9B5,  BGW9C3 |

The soil types were classified using the GPS coordinates information of the sites and the World Reference Base classification system (IUSS Working Group WRB., 2014).

**Table S2_** **supplementary data. Information of nucleotide sequence used in the phylogenetic analysis.**

| **Locus** | **Number of strains** | **Nucleotide sequence information** | | | | |  |
| --- | --- | --- | --- | --- | --- | --- | --- |
|  |  | **Distinct pattern** | **Parsimony-informative** | **Best-fit Model** | **Conserved site** | ***Total** | |
| *atpD* | 107 | 120 | 105 | TIM2+F+I+G4 | 212 | 337 | |
| *dnaK* | 109 | 106 | 92 | TIM2+F+G4 | 115 | 228 | |
| *glnII* | 97 | 155 | 128 | TIM2+F+I+G4 | 244 | 402 | |
| *gyrB* | 93 | 191 | 189 | GTR+F+I+G4 | 197 | 419 | |
| *recA* | 122 | 150 | 134 | TIM2+F+I+G4 | 222 | 375 | |
| *rpoB* | 90 | 150 | 147 | TIM+F+G4 | 184 | 366 | |
| *nodA* | 90 | 474 | 378 | TPM3+F+I+G4 | 210 | 636 | |
| *nifH* | 68 | 382 | 280 | TIM2+F+I+G4 | 560 | 888 | |
| *atpD-dnaK-glnII-gyrB-recA -rpoB* | 89 | 766 | 783 | # | 1182 | 2127 | |

*Number of sites used for tree construction

#A partition model was used

**Table S3_ supplementary data. Distribution of genospecies (GS) and *recA* groups (rA) in sampling sites.**

| Location | GSI | GSII | GSVII | GSVIII | GSIX | GXII | Location | rAII | rAIII | rAIV | rAVI | rAVIII | rAXII | rAXIII |
| --- | --- | --- | --- | --- | --- | --- | --- | --- | --- | --- | --- | --- | --- | --- |
| Tansia | 1 | 1 | 0 | 0 | 0 | 0 | Tansia | 0 | 0 | 0 | 0 | 3 | 0 | 0 |
| Kasiesa | 1 | 1 | 0 | 0 | 0 | 0 | Kasiesa | 2 | 0 | 0 | 0 | 1 | 0 | 0 |
| Bogrigo | 0 | 0 | 2 | 0 | 0 | 0 | Bogrigo | 0 | 2 | 0 | 0 | 0 | 0 | 0 |
| Belempiisi | 1 | 0 | 0 | 1 | 0 | 0 | Belempiisi | 2 | 0 | 2 | 0 | 0 | 1 | 0 |
| Manga | 0 | 0 | 0 | 2 | 0 | 0 | Manga | 0 | 0 | 0 | 0 | 1 | 4 | 1 |
| Golinga | 1 | 1 | 0 | 2 | 0 | 0 | Golinga | 0 | 0 | 5 | 0 | 2 | 3 | 0 |
| Upando | 0 | 0 | 2 | 0 | 0 | 0 | Upando | 0 | 8 | 0 | 0 | 0 | 0 | 0 |
| Masaka | 3 | 0 | 1 | 2 | 0 | 0 | Masaka | 1 | 4 | 6 | 0 | 0 | 2 | 0 |
| Changnaayili | 0 | 0 | 0 | 1 | 1 | 0 | Changnaayili | 0 | 0 | 0 | 0 | 0 | 1 | 2 |
| Mepasem | 0 | 0 | 2 | 0 | 0 | 0 | Mepasem | 0 | 3 | 0 | 0 | 0 | 0 | 0 |
| Gunayili | 0 | 0 | 0 | 0 | 0 | 3 | Gunayili | 0 | 0 | 2 | 2 | 0 | 0 | 0 |
| Achubonyor | 1 | 1 | 0 | 0 | 0 | 0 | Achubonyor | 0 | 2 | 4 | 0 | 0 | 0 | 1 |
| Nabori | 0 | 0 | 1 | 0 | 0 | 0 | Nabori | 0 | 1 | 0 | 0 | 0 | 0 | 0 |
| Loho | 2 | 1 | 0 | 0 | 0 | 0 | Loho | 0 | 0 | 3 | 0 | 5 | 0 | 0 |
| Tanina | 0 | 0 | 1 | 0 | 0 | 1 | Tanina | 0 | 2 | 1 | 1 | 0 | 0 | 0 |
| Bamahu | 0 | 0 | 1 | 0 | 1 | 0 | Bamahu | 0 | 1 | 1 | 0 | 0 | 0 | 0 |
| Gbanko | 0 | 1 | 0 | 1 | 1 | 0 | Gbanko | 0 | 0 | 0 | 0 | 4 | 1 | 1 |
| Sanwana | 2 | 0 | 0 | 0 | 0 | 0 | Sanwana | 0 | 0 | 2 | 0 | 0 | 0 | 0 |
| Kunfabiala | 0 | 0 | 1 | 0 | 0 | 0 | Kunfabiala | 0 | 5 | 1 | 0 | 0 | 0 | 0 |
| Sum | 12 | 6 | 11 | 9 | 3 | 4 | Sum | 5 | 28 | 27 | 3 | 16 | 12 | 5 |

**Table S4_ supplementary data. Accession numbers of reference strains.**

| **STRAIN** | ***recA*** | ***atpD*** | ***dnaK*** | ***glnII*** | ***gyrB*** | ***rpoB*** |
| --- | --- | --- | --- | --- | --- | --- |
| *B. algeriense* RST89^T^ | FJ264927.1 | KF956544.1 | FJ264922.1 | FJ264924.1 | NA | NA |
| *B. americanum* CMVU44^T^ | KC247141 | KC247125.1 | NA | KX012942 | NA | NA |
| *B. amphicarpaeae* 39S1MB^T^ | KF615002.1 | KP768547 | CP029426.1 | KP768605 | KP768721.1 | KP768663 |
| *B. arachidis* CCBAU 051107^T^ | HM107233.1 | HM107217.1 | FPBQ01000001.1 | HM107251.1 | KF962693 | JX437682 |
| *B. betae* PL7HG1^T^ | FJ970378.1 | CP044543 | AY923046.1 | FJ970431.1 | CP044543.1 | GU562860.1 |
| *B. brasilense* UFLA03-321^T^ | KT793142 | KF452730.1 | KF452791.1 | NA | KF452827 | KF452879.1 |
| *B. cajani* AMBPC1010^T^ | KY349440 | WQNE01000013 | WQNE01000019.1 | KY349443 | WQNE01000054 | WQNE01000005.1 |
| *B. canariense* BTA-1^T^ | FM253177.1 | FM253135 | AY923047.1 | AY386765 | FM253220 | FM253263 |
| *B. centrolobii* BR 10245^T^ | KX527954.1 | LUUB01000107 | LUUB01000108 | KX527991.1 | KX528004.1 | KF983827.3 |
| *B. centrosematis* A9^T^ | KC247145 | KC247129.1 | NA | KX012940 | NA | NA |
| *B. cytisi* CTAW11^T^ | GU001575.1 | LM994389.1 | LM994142.1 | GU001594.1 | KF532653.1 | LM994166.1 |
| *B. daqingense* CCBAU 15774^T^ | HQ231270.1 | HQ231289 | LM994144.1 | HQ231301.1 | LM994190.1 | JX437676 |
| *B. denitrificans* IFAM 1005^T^ | FM253196.1 | FM253153 | KF962685.1 | HM047121 | FM253239 | FM253282 |
| *B. diazoefficiense* USDA 110^T^ | CP011360 | CP011360 | CP011360 | CP011360 | CP011360 | CP011360 |
| *B. elkanii* USDA 76^T^ | AY591568 | KB900701.1 | KB900701.1 | AY599117 | AB070584.1 | KB900701.1 |
| *B. embrapense* CNPSo 2833^T^ | HQ634899.1 | HQ634875.1 | KP234519.2 | GQ160500.1 | HQ634891.1 | HQ634910.1 |
| *B. erythrophlei* CCBAU 53325^T^ | KF114669.1 | NA | MG811656.1 | KF114693 | KF114717.1 | MG811654.1 |
| *B. ferriligni* CCBAU 51502^T^ | KJ818112 | NA | MG811657.1 | KJ818099 | KJ818102.1 | MG811655.1 |
| *B. forestalis* INPA54B^T^ | KF452867.1 | KF452722.1 | PGVG01000013.1 | NA | KF452831.1 | PGVG01000026 |
| *B. frederickii* CNPSo 3426^T^ | MK682710.1 | SPQS01000024 | SPQS01000014.1 | MK682688.1 | MK682721.1 | SPQS01000016 |
| *B. ganzhouense* RITF806^T^ | JX277144 | JX277182.1 | NA | JX277110 | NA | NA |
| *B. guangdongense* CCBAU 51649^T^ | KC509269.1 | KC508916.1 | CP030051.1 | KC509023.1 | KC509072.1 | KC509318.1 |
| *B. guangxiense* CCBAU 53363^T^ | KC509279.1 | KC508926.1 | CP022219.1 | CP022219 | KC509082.1 | KC509328.1 |
| *B. guangzhouense* CCBAU 51670^T^ | KC509254.1 | KC508902.1 | CP030053.1 | KC509008.1 | KC509057.1 | KC509303.1 |
| *B. huanghuaihaiense* CCBAU 23303^T^ | HQ231595.1 | HQ231682 | LM994145.1 | HQ231639.1 | LM994191.1 | HQ428068.1 |
| *B. icense* LMTR 13^T^ | JX943615 | KF896192 | KF896182.1 | KF896175 | KF896201 | CP016428 |
| *B. ingae* BR 10250^T^ | KF927061 | KY753593.1 | KF927055.1 | KF927067 | KF927079.1 | KF927073.1 |
| *B. iriomotense* EK05^T^ | AB300996 | LM994395.1 | LM994146.1 | AB300995 | AB300997.1 | LM994170.1 |
| *B.* *ivorense* CI-1B^T^ | MK376330.1 | CAADFC020000004 | MK376326.1 | MH756157.1 | MH756161.1 | CAADFC020000027 |
| *B. japonicum* USDA 6^T^ | NC_017249.1 | AM168320 | NC_017249.1 | HQ587875 | MH756161.1 | LC167354.1 |
| *B. jicamae* PAC68^T^ | HM590776.1 | FJ428211 | LM994149.1 | FJ428204 | JN685764.1 | LM994173.1 |
| *B. kavangense* 14-3^T^ | KM378399 | KY753592 | KR259949.1 | KM378446 | KX661397 | KM378311 |
| *B. lablabi* CCBAU 23086^T^ | GU433522.1 | GU433473 | LM994147.1 | GU433498 | LM994192.1 | JX437677 |
| *B. liaoningense* 2281^T^ | FM253180.1 | FM253137 | AY923041.1 | AY386775 | FM253223 | FM253266 |
| *B. lupini* USDA 3051^T^ | MN525209.1 | KU738808.1 | MN525206.1 | NA | MK689366.1 | MN525210.1 |
| *B. macuxiense* BR 10303^T^ | KX527958.1 | LNCU01000024 | KX527932.1 | KX527995.1 | KX528008.1 | KX527969.1 |
| **Table S4_ supplementary data continued.** | | | | | | |
| **STRAIN** | ***recA*** | ***atpD*** | ***dnaK*** | ***glnII*** | ***gyrB*** | ***rpoB*** |
| *B. manausense* BR 3351^T^ | KF785992.1 | LJYG01000004 | KF786001.1 | KF785986.1 | KF786000.1 | LJYG01000045 |
| *B. mercantei* SEMIA 6399^T^ | KX690615 | MKFI01000006 | KX690617.1 | KX690622.1 | KX690623.1 | MKFI01000001 |
| *B. namibiense* 5-10^T^ | KM378377 | KX661387 | KP402058.1 | KM378440 | KX661393 | KM378306 |
| *B. nanningense* CCBAU 53390^T^ | KC509274.1 | KC508921.1 | LBJC01000024 | KC509028.1 | KC509077.1 | KC509323.1 |
| *B. neotropical* BR 10247^T^ | KJ661714.1 | LSEF01000046 | KJ661693.1 | KJ661700 | KJ661707.1 | KF983829 |
| *B. niftali* CNPSo 3448^T^ | MK675797.1 | SPQT01000036 | SPQT01000034 | MK675791.1 | MK675794.1 | MK675800.1 |
| *B. nitroreducens* TSA1^T^ | LFJC01000003 | LFJC01000003 | LFJC01000003.1 | NA | LFJC01000003 | LFJC01000003 |
| *B. oligotrophicum* S58^T^ | JQ619231 | JQ619232 | KF962688.1 | JQ619233 | KF962697 | KF962713 |
| *B. ottawaense* OO99^T^ | HQ587287 | HQ455212 | JF308816.1 | HQ587750 | HQ873179 | HQ587518 |
| *B. pachyrhizi* PAC48^T^ | HM590777.1 | FJ428208 | LM994148.1 | FJ428201 | KF532651.1 | LM994172.1 |
| *B. paxllaeri* LMTR 21^T^ | JX943617.1 | KF896186.1 | AY923038.1 | (KF896169.1 | KF896195.1 | KP308154.1 |
| *B. retamae* Ro19^T^ | KC247094.1 | KC247101 | LM994150.1 | KC247108 | KF896204.1 | KF962714 |
| *B. rifense* CTAW71^T^ | GU001585.1 | GU001617 | LM994143.1 | GU001604.1 | KC569466.1 | KF962715 |
| *B. ripae* WR4T | MF593090 | NA | MF593102.1 | MF593086.1 | MF593094.1 | MF593098.1 |
| *B. sacchari* BR 10280^T^ | KX065095 | KX065107 | KX065103.1 | KX065099.1 | LWIG01000001 | LWIG01000014 |
| *B. shewense* ERR11^T^ | JQ809837.1 | FMAI01000019 | FMAI01000013.1 | JQ809893.1 | FMAI01000013 | JQ810006.1 |
| *B. stylosanthis* BR 446^T^ | KU724163.1 | LVEM01000002 | KU724145.1 | KU724148.1 | KU724151.1 | KU724166.1 |
| *B*. *subterraneum* 58 2-1^T^ | KM378397 | KX661391.1 | KP308157.1 | KM378484.1 | KX661396.1 | KM378349.1 |
| *B. symbiodeficiens* 85S1MB^T^ | KF615036 | KP768551 | CP029427.1 | KP768609 | KP768725 | KP768667 |
| *B. tropiciagri* CNPSo 1112^T^ | FJ391168.1 | FJ390968.1 | FJ391008.1 | FJ391048.1 | HQ634890.1 | HQ634909.1 |
| *B. valentinum* LmjM3^T^ | JX518589.2 | JX518561.2 | LLXX01000028.1 | JX518575.1 | LLXX01000044 | LLXX01000029 |
| *B. vignae* 7-2^T^ | KM378374.1 | RDQF01000079 | KR259951.1 | KM378443.1 | KR149134.1 | KM378308.1 |
| *B. viridifuturi* SEMIA 690^T^ | KR149140.1 | LGTB01000039 | KR149128.1 | KR149131.1 | KR149134.1 | KU724169.1 |
| *B. yuanmingense* CCBAU 10071^T^ | AY591566.1 | AY386760.1 | AY923039.1 | AY386780 | HE576508.1 | FMAE01000008 |
| *B. zhanjiangense* CCBAU 51778^T^ | KC509263.1 | KC508911.1 | KX683404.1 | KC509017.1 | KC509066.1 | KC509312.1 |

NA, sequences not available
